# Exosomes as Naturally Occurring Vehicles for Delivery of Biopharmaceuticals: Insights from Drug Delivery to Clinical Perspectives

**DOI:** 10.3390/nano11061481

**Published:** 2021-06-03

**Authors:** Arun Butreddy, Nagavendra Kommineni, Narendar Dudhipala

**Affiliations:** 1 Formulation R&D, Biological E. Limited, IKP Knowledge Park, Shameerpet, Hyderabad 500078, Telangana State, India; butreddyarun@gmail.com; 2 College of Pharmacy and Pharmaceutical Sciences, Florida A&M University, Tallahassee, FL 32307, USA; nagavendra.kommineni@famu.edu; 3 Depratment of Pharmaceutics, Vaagdevi College of Pharmacy, Warangal 506005, Telangana State, India

**Keywords:** exosomes, drug delivery, isolation, characterization, stabilization, route of administration

## Abstract

Exosomes as nanosized vesicles are emerging as drug delivery systems for therapeutics owing to their natural origin, their ability to mediate intercellular communication, and their potential to encapsulate various biological molecules such as proteins and nucleic acids within the lipid bilayer membrane or in the lumen. Exosomes contain endogenous components (proteins, lipids, RNA) that could be used to deliver cargoes to target cells, offering an opportunity to diagnose and treat various diseases. Owing to their ability to travel safely in extracellular fluid and to transport cargoes to target cells with high efficacy, exosomes offer enhanced delivery of cargoes in vivo. However, several challenges related to the stabilization of the exosomes, the production of sufficient amounts of exosomes with safety and efficacy, the efficient loading of drugs into exosomes, the clearance of exosomes from circulation, and the transition from the bench scale to clinical production may limit their development and clinical use. For the clinical use of exosomes, it is important to understand the molecular mechanisms behind the transport and function of exosome vesicles. This review exploits techniques related to the isolation and characterization of exosomes and their drug delivery potential to enhance the therapeutic outcome and stabilization methods. Further, routes of administration, clinical trials, and regulatory aspects of exosomes will be discussed in this review.

## 1. Introduction

Over decades, synthetic drug delivery systems such as liposomes, micelles, dendrimers, and polymeric nanoparticles have been exploited to improve the efficacy and therapeutic index (in terms of the pharmacokinetics and pharmacodynamics profiles) of therapeutics, while minimizing the toxicity and drug-related off-target side effects [1,2,3,4]. Many hurdles still exist for synthetic drug delivery systems, including the delivery of drugs to target organs, toxicity owing to the chemical and physical features of the synthetic delivery system, reactions to the host immune system, and activation of an acute hypersensitivity reaction, which can result in discontinuation of treatment in some individuals. The utilization of extracellular vesicles as a natural carrier system to deliver therapeutics can overcome the limitations associated with synthetic drug delivery systems [1,5,6,7].

**Figure 1:**
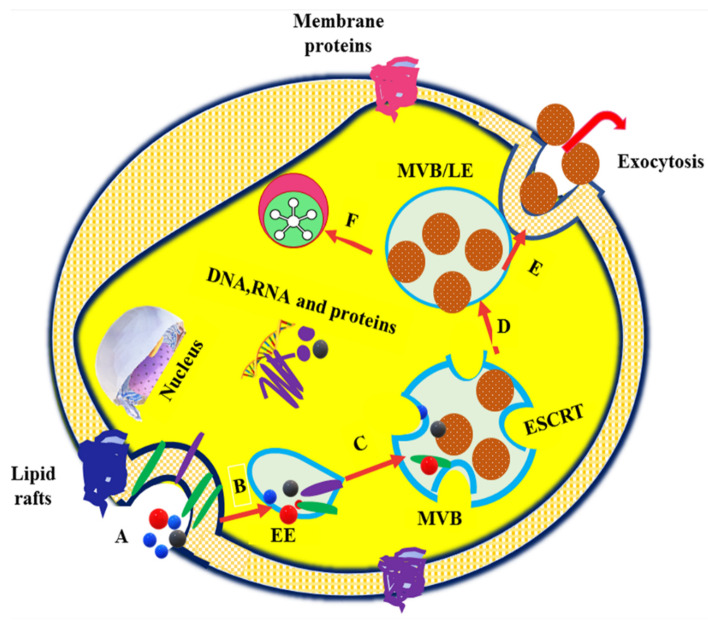
Exosome vesicle formation, cargo sorting (starts from endocytosis and ends in the MVBs), and release. (**A**). Endocytosis of the plasma membrane (**B**) Uptake of proteins, nucleic acids, and membrane-associated molecules into encysted body and formation of EE. (**C**) Transformation of early endosomes (EE) into multivesicular bodies (MVB) or late endosomes (LE), intraluminal vesicle (ILV) formation via inward budding of MVB or LE, and cargo sorting through ESCRT- and ESCRT-independent pathways. (**D**) Fusion of MVB or LE with the plasma membrane and lysosome. (**E**) Exocytosis of exosomes in response to MVB–plasma membrane interactions. (**F**). Degradation of MVB or LE by the lysosome (modified from [14]).

Extracellular vesicles are differentiated into apoptotic bodies, microvesicles, and exosomes, depending on the intracellular origin and size. Apoptotic bodies contain cellular contents, including deoxyribonucleic acid (DNA), ribonucleic acid (RNA), and histone proteins, with sizes ranging from 50 to 5000 nm and which are formed by membrane blebbing during apoptosis. Microvesicles, also known as microparticles or ectosomes, have sizes ranging from 50 nm to 1000 nm and are formed through fission from plasma membranes. Exosomes are nanosized membrane vesicles with a size range of 30–100 nm, which are secreted by various types of cells [8,9,10,11]. Exosome formation typically involves the formation of endocytic vesicles from the plasma membrane; inward budding of the endosomal vesicle membrane, which results in the multivesicular body (MVB); and the release of exosomes derived from the MVB into the extracellular environment when the MVB fuses with the plasma membrane [12]. The exosome biogenesis, cargo sorting, and vesicle release processes are shown in Figure 1. Owing to their nanoscale dimensions, exosomes have received greater attention in recent years and are considered to be the most promising vehicles for drug delivery to target cells or organ [13]. 

Exosomes are released by cells in both physiological and pathological circumstances. As exosomes circulate in the blood, they may operate as signal transducers both locally and far away from their source [15]. For example, exosomes are released via activation at sites of vascular damage, where they may have a signaling or adhesion function. Antigen-presenting cells also secrete exosomes that carry peptide-loaded MHC molecules functioning as intercellular vehicles for antigenic materials [16,17]. Several studies have reported that exosomes have been found to be released from several regions of the female reproductive system, including the endometrium, uterus, oviduct epithelium, placenta trophoblastic cells, and preimplantation embryos. Exosomes have critical roles in modulating transcription and translational activity, granulosa cell proliferation and differentiation, cumulus expansion, gametogenesis, proper follicular growth, oocyte maturation, fertilization rate regulation, embryo development, and blastocyst formation and implantation, as well as pregnancy outcomes and fertility [18,19,20,21,22,23]. The presence of exosomes in reproductive system secretions further indicates their potential functions in preconception and postconception intercellular interactions; nonetheless, modified exosomes have recently been employed as markers for pregnancy and diseases linked with pregnancy in humans [23,24,25].

Exosomes are involved in sperm activities and epigenetic inheritance and are secreted by the epididymis (epididymosomes) and prostate (prostasomes). There are two types of epididymosomes: CD9+ epididymosomes and ELSPBP1-enriched epididymosomes. The CD9+ epididymosomes govern sperm maturation by transferring their protein cargoes to the spermatozoa. ELSPBP1-enriched epididymosomes bind to dead spermatozoa preferentially, quenching reactive oxygen species that could otherwise harm sperm maturation [26]. Protein cargoes in prostasomes, as with those in epididymosomes, are transported to spermatozoa and are involved in sperm survival and motility via calcium-dependent signaling. Despite the fact that the prostasome contains DNA, coding, and regulatory RNAs with potential modulatory activities, there is little indication that these nucleic acids are transferred to spermatozoa [27]. Choy et al. isolated and characterized the testicular exosomes, demonstrating that they were taken up by somatic and germ cells, including sperm cells. Their findings have provided new insights into intercellular communication in the testes, which have broad implications for spermatogenesis and paternal epigenetic inheritance [28]. Proteins in the exosomes may be associated with cell recognition, allowing them to target a certain cell type. The majority of epididymosome-associated proteins are transferred to the subcellular or membranous sperm domains during epididymal transit and are involved in the acquisition of fertilization ability, modulation of motility, and protection against oxidative stress. Proteins associated with prostasomes stimulate sperm motility and regulate the capacitation timing to prevent premature acrosome response induction [29]. In a previous study, Choy et al. revealed the plethora of proteins in the testicular exosomes that have been implicated in male fertility, suggesting that the communication mediated by testicular exosomes is required for spermatogenesis [28].

Exosomes play an essential role in intercellular communication by carrying genetic and proteomic information between neighboring cells or distant organs. Exosomes communicate their message or deliver the cargo to the recipient cell in different ways. Firstly, exosomes bind to target cell membranes through ligands expressed on their surfaces, facilitating the ligand–receptor interaction. This ligand–receptor interaction elicits an immune response and mediates hemostasis, angiogenesis, and cancer progression [30]. Secondly, exosomes transfer their surface proteins and cytoplasms to the target cells through budding and subsequent fusion with the plasma membranes of the target cells [31,32]. The third mechanism involves the horizontal transfer of proteins and genetic material from one cell to another. Studies have shown that fusion or internalization of exosomes can aid in the transfer and release of their cargo and in mediating regulatory processes [8,33]. Despite the wide range of functions carried out by exosomes, little is known about the molecular pathways involved in exosome secretion. Exosome release is aided by the presence of Ca^2+^. In most cell types, an increase in the intracellular Ca^2+^concentration, a universal intracellular signal, is required to initiate exosome secretion. During the exocytosis process, the membrane of a secretory vesicle fuses with the plasma membrane in a tightly controlled Ca^2+^-triggered reaction. In endocrine cells, secretory granules contain many Ca^2+^ ions, and it has been suggested that a high intragranular Ca^2+^ concentration is required for effective exocytosis [17]. Therefore, it is evident that many intracellular transport events depend on Ca^2+^, and thus it is likely that Ca^2+^ might be required for the fusion events involved in the secretion of the exosomes [34].

Exosomes have many properties that a drug delivery vehicle should have, such as tolerability due to their wide distribution in biological fluids, the ability to exert functional responses by transferring their cargoes across the membranes of the target cells, the potential to mediate intercellular transfer of mRNAs and miRNAs, and the ability to cross biological barriers [35,36,37]. Various cell types are used to obtain exosomes, including mesenchymal cells, immune cells, and tumor cells. The selection of the cell type is crucial because the exosomes’ functions (quantity of drug load, amount of exosome release) will depend on the properties of the cell types. Moreover, the biodistribution of exosomes may vary depending on the cell origin [38].

In exosome formulation, biomolecules including coding and non-coding RNAs and cell-targeting and cell adhesion moieties are packed within the lumen or lipid bilayer. The exosome structure is similar to the unilamellar liposome, whereby an amphiphilic lipid bilayer surrounds an aqueous core. Due to this structural resemblance, there are expected to be similar characteristics between exosome and liposome drug delivery systems [11]. However, the low immunogenicity, non-cytotoxicity, and non-mutagenicity of exosomes give them superior drug delivery potential compared to liposomes [39]. For these reasons, exosomes can be used as specific drug delivery systems by selecting the exosome source. Exosomes possess different roles, acting as innate bio-therapeutics, therapeutic targets, and drug delivery carriers. Several approaches can be used to maximize the efficacy of exosomes, including introducing exogenous drug molecules, increasing their innate therapeutic capability, and altering their surfaces to improve their in vivo bio-distribution and attenuate their pathological activities [40]. 

**Figure 2:**
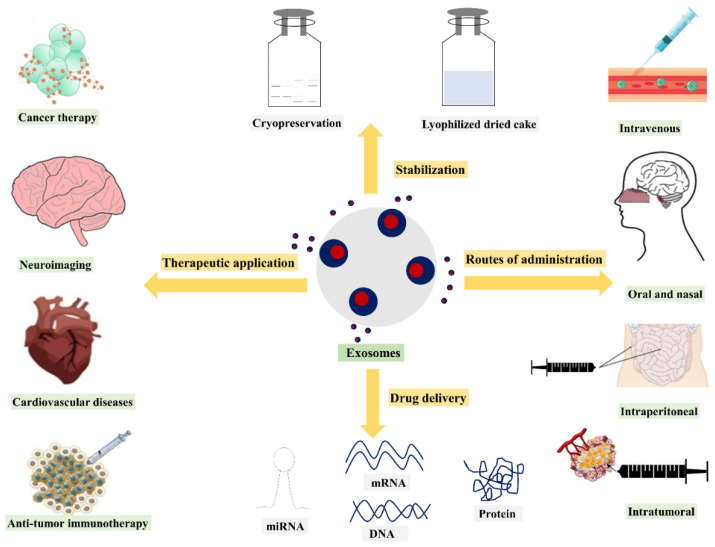
Overview of drug delivery and therapeutic applications of exosomes.

To date, researchers have mainly utilized exosomes as drug or gene carriers, disease markers, and therapeutic targets. In recent years, various methods have been used for exosome isolation and characterization. Numerous studies have demonstrated that depending on the different cell types, exosomes containing different compositions exhibit different functions. As a result, the current review discusses exosome drug delivery systems from the following perspectives: exosome isolation or purification and characterization methods; exosome applications as drug, gene, and nucleic acid delivery systems; the various administration routes of exosomes, as well as their production methods and scalability challenges. Additionally, clinical trials and regulatory challenges related to exosome delivery systems will be reviewed and discussed. An overview of the use of exosomes for drug delivery, their clinical applications, and their potential administration routes with stabilization strategies is presented in Figure 2.

## 2. Isolation or Purification Methods

To obtain ultrapure exosomes, it is imperative to isolate exosomes from cell fragments and interfering substances. Different techniques can be employed to isolate or separate exosomes from cell culture or body fluids, depending on the exosome source and size. Various techniques have been developed to isolate exosomes, including differential centrifugation, filtration, size exclusion chromatography (SEC), and polymer precipitation techniques. Table 1 shows the strategies, mechanisms, advantages, and disadvantages of exosome isolation techniques.

### 2.1. Ultracentrifugation

Ultracentrifugation is referred to as the classic and golden standard method used to isolate exosomes. This method utilizes centrifugal force to condition cell culture media or biological fluids to remove cells and large cell debris according to the density, size, and shape [41]. Théry et al. reported an experimental protocol for ultracentrifugation to obtain and isolate exosomes, which is as follows: (1) The culture-conditioned medium is initially centrifuged at 300× *g* for 10 min to separate or remove the living cells. (2) The collected supernatant is then centrifuged to precipitate the dead cells at a centrifugation force of 2000× *g* for 10 min; (3) The collected supernatant is centrifuged at 10,000× *g* for 30 min to eliminate the cell debris, then the collected supernatant is centrifuged at 100,000× *g* for 70 min to precipitate the exosomes. The pellet collected is washed with a large volume of phosphate-buffered saline (PBS) to remove the contaminated proteins. (4) The resultant solution is finally centrifuged at 100,000× *g* for 70 min to obtain an ultrapure exosome [42]. With the continuous development of centrifugation technology, combining ultracentrifugation with density gradient separation could significantly enhance exosome isolation [43]. Although ultracentrifugation is an established method used to isolate exosomes, repeated use of this method can rupture the exosome membrane due to the effects of centrifugal force on the exosome [44].

### 2.2. Density Gradient Ultracentrifugation

This method is based on increasing the density gradient of solutions from the top of the tube to the bottom. After the centrifugation, contaminants with densities different than exosomes will be separated into layers, while the exosomes will sediment into other layers matching the density of the exosomes [11]. Sucrose, iodixanol in water, and ice-cold PBS are the most commonly used gradient media for exosome isolation [45]. By using the gradient density of sucrose, contaminants whose density is different than the exosome density may be separated from the exosomes, thereby producing a theoretically pure fraction of exosomes. Density gradient ultracentrifugation is considered one of the best methods due to the purity, yield, and preservation of vesicular structure when compared to other physical exosome isolation methods [46]. However, contamination of the exosome-containing fraction with high-density and low-density lipoproteins has been observed [47]. The exosomes isolated using ultracentrifugation may interfere with protein aggregates, apoptotic bodies, and other non-exosome microvesicles. This can be overcome by using density gradient ultracentrifugation [48].

### 2.3. Ultrafiltration

Exosomes can be separated from cell debris, soluble protein aggregates, and other extracellular vesicles using standard membrane filters with defined molecular weight or size exclusion limits. As exosomes are small, they can be isolated according to their size. Ultrafiltration is typically used as a subsequent step during ultracentrifugation and as a final step in chromatography. The typical protocol for ultrafiltration is as follows: (1) dead-end filtration to separate the floating cells and cell debris from the cell culture supernatant using a 0.1 µm filter at 22 °C temperature; (2) tangential flow filtration (TFF) of the filtrate through a filter with a molecular weight cutoff of 500 kDa at a process temperature of 4 °C; (3) further filtration of the deposits from step 2 using a sterilized 100 nm filter [49].

Ultrafiltration can be performed using either direct flow filtration or tangential flow filtration. Direct filtration, also known as dead-end filtration, is most preferred for filtration of small-volume samples of up to 30 mL, however it has problems related to membrane fouling and impaired particle separation [50,51]. Tangential flow filtration, also known as crossflow filtration, is a more efficient, convenient, and rapid process to isolate exosomes on a large-scale basis. In TFF, the sample fluid flows tangentially through the filter membrane to avoid clogging or cake formation. Briefly, samples filtered through a 0.2 µm polyethersulfone membrane are subjected to the ultrafiltration process through a TFF system with a cartridge filter membrane (500 kDa molecular weight cutoff) at a flow rate of 120 mL/min, transmembrane pressure of <3.5 psi, and crossflow rate of >10:1 [50]. Exosomes that are too large to pass through the membrane pores remain as a retentate, whereas small molecules including free proteins are eluted as a permeate after passing through hollow fiber pores and ultimately discarded from the process. Further, the retentate is reconcentrated serially by TFF to deplete the contaminants smaller than 500 kDa and the purified exosomes are resuspended and stored in 0.1 M sucrose at −80 °C [49]. Ultrafiltration can be combined with other techniques such as chromatography to enhance the exosome purity after isolation using ultrafiltration [52].

### 2.4. Size Exclusion Chromatography (SEC)

In SEC, large molecules and other particulate matter are separated by utilizing a porous stationary phase, depending on the exosome size. The small hydrodynamic radius components in the sample of interest can pass through the pores, resulting in late elution. In contrast, it is difficult for components with a relatively larger hydrodynamic radius (exosomes) to enter the pores, leading to early elution [53]. It has been reported that exosomes isolated from the SEC can maintain the structure of the exosomes, as confirmed by transmission electron microscopy. Moreover, the structure and integrity of the exosomes during isolation from the SEC will not be affected by shear force compared to the centrifugal method. This is because SEC can be performed under low pressure, which maintains the integrity of exosomes during isolation. This technique mainly helps to separate or remove the protein or lipoprotein impurities from the isolated exosomes. Additionally, this method has been used as a subsequent isolation technique for ultrafiltration and ultracentrifugation methods [14]. However, the relatively long running time limits the use of the SEC technique in the isolation of samples [54]. 

### 2.5. Precipitation Technique

This technique involves charge-based precipitation of exosomes by utilizing hydrophilic polymers such as polyethylene glycol (PEG). PEG causes a decrease in the solubility of exosomes by hijacking the water molecules and making exosomes settle under low-speed centrifugal conditions. Briefly, co-incubation of exosome-containing samples with a solution of PEG (molecular weight of 8000 Da) leads to precipitation of the exosomes. The precipitated exosome samples can be recycled or separated using either filtration or centrifugation when incubated at 4 °C overnight. This method does not require specialized equipment and is relatively easy to operate without lengthy running times [54,55]. Kanchi et al. [56] reported the potential application of the precipitation technique to isolate the exosomes from urinary fluid using a DL–dithiothreitol solution to remove or separate the polymeric networks of Tamm–Horsfall protein. Subsequent precipitation of exosomes was performed at 25 °C at a centrifugal force of 10,000× *g* for 30 min. The recovery and resuspension of exosomes isolated by the precipitation method is more efficient than ultracentrifugation-based isolation [57].

### 2.6. Immunoisolation

The immunoisolation or immunoaffinity technique uses magnetic beads coated with antibodies to identify certain proteins on the lipid bilayer membrane of the exosomes, thereby separating them from other substances [58,59]. It has been reported that biomarkers such as CD34, CD63, and CD326 are often used as the biomarkers of acute myeloid leukemia blasts, human exosomes, and tumor exosomes, respectively [60,61]. An immunoaffinity isolation kit (microplate-based enzyme linked immunosorbent assay, ELISA) can be used to isolate the exosomes based on the exosome surface markers and tetraspanin proteins, considered as determining factors for the immunoisolation technique [14]. Compared to ultracentrifugation, the immunoisolation method is superior in capturing a small quantity of plasma with high specificity. Thus, it is often used to further isolate the specific exosomes that have been previously isolated by other techniques. Nevertheless, this technique is applicable for the isolation of exosomes that are specific to the particular biomarkers [41,62]. 

### 2.7. Microfluidics-Derived Chip Isolation Methods

In recent years, the microfluidics-based chip isolation techniques have become a promising approach for the separation of exosomes. These methods are based on the difference between the physical and biochemical properties of the exosomes, such as the size, density, and immunoaffinity. The purification and isolation methods utilizing microfluidics-based chip isolation techniques can be categorized into three approaches, namely approaches involving immunoaffinity for exosome trapping, sieving approaches, and approaches involving exosomes being adsorbed into the porous structure [63]. All three approaches require off-chip steps for sample preparation, such as reagent mixing and plasma extraction, which enhance the processing complexity. Exosomes measuring around 40–100 nm in size are specifically entrapped with this technique, and the specificity of the exosomes is high, particularly for the microfluidic-chip-based immunoaffinity capture approach. The sieving method can be used to separate the exosomes from the whole blood based on pressure or electrophoresis [14,64]. Low cost, portability, and fast sample processing are some of the advantages of this technique. However, the ability to efficiently separate, purify, and economically produce the exosomes in sufficient quantities may limit the entry of this technique into the clinical market [54].

**Table 1:** Strategies, advantages, and disadvantages of exosome isolation or purification methods.

Isolation or Purification Methods	Mechanism	Advantages	Limitations	References
Ultracentrifugation	Sedimentation coefficient of exosomes and other substances in a sample.	Capable of producing a large number of exosomes with high separation purity.	Poor repeatability, low recovery rate, requires more time, unsuitable for clinical diagnosis.	[65,66,67]
Density gradient ultracentrifugation	Separation based on the different densities.	Preserves exosome vesicle integrity, while yield can be maximized for samples that are already pure.	Difficult to scale-up, multi-step procedure.	[11,44,68]
Ultrafiltration	Separation based on size and molecular weight.	Faster, requires no special equipment, easy to handle compared to ultracentrifugation.	Deformation and breaking up of large vesicles may occur due to the use of force.	[69,70]
Size exclusion liquid chromatography	Utilizes a column packed with porous polymeric beads, which separate the exosomes based on size.	Allows separation of large and small molecules. The structure of the exosomes isolated by this method is not affected by shearing force compared to centrifugation methods.	Requires a long running time, which limits the processing of multiple biological samples.	[71,72]
Immunoaffinity capture-based techniques	Based on the specific interactions between immobilized antibodies (ligands) and membrane-bound antigens (receptors) of exosomes.	Suitable for isolation of specific exosomes, high possibility of subtyping, high-purity isolation.	High reagent cost, low capacity and yield, cannot be used for the separation of exosomes at a large scale. Requires non-physiological salt and pH conditions.	[66,73]
Precipitation technique	Change in the dispersibility or solubility of exosome vesicles using water excluding polymers.	Easy to use, requires no specialized equipment, and scalable sample capacity.	Requires long running times and preand postseparation cleanup. Co-precipitation of non-exosome contaminants within the sample.	[74,75,76]
Microfluidic technologies	Immunoaffinity, sieving, and trapping of exosomes on porous structure.	Quantitative technique that allows high-throughput analysis of exosome contents with high sensitivity.	Low sample capacity. Lack of standardization, method validation, and large-scale tests on clinical samples.	[70,77]

## 3. Exosomes in Drug Delivery

Typically, liposomes and polymeric nanoparticles are the most preferred drug delivery systems for entrapment or encapsulation of drug molecules, and these delivery systems are routinely used to deliver different classes of drug molecules, including anticancer, antifungal, and analgesics molecules. However, the biocompatibility, better stability, long-term safety, and ability to evade the host immune system with long systemic circulating capability and stability remain major concerns [6,12].

Over the past few years, considerable efforts have been made to develop exosomes as novel nanoscale delivery systems. Several characteristics of exosomes, including their biocompatiblity and biodegradability, mean they do not elicit acute immune reactions, have low toxicity, carry the required fusogenic properties, contain low-uptake machinery, have high specificity to the target cells, and are smaller in size, making exosomes attractive nanocarrier drug delivery systems. Further, exosomes have a tendency to accumulate more in tumor tissues than in normal tissue. Moreover, the specificity of the exosome delivery platform can be further enhanced by anchoring exosomes with tumor targeting ligands such as proteins, peptides, or antibodies for specifically targeted drug delivery [78,79]. Table 2 shows the application of exosomes for therapeutic delivery in various pathological conditions.

### 3.1. Protein and Peptide Delivery:

Exosomes represent a favorable therapeutic approach for delivering protein or peptide molecules. Initially, exosomes were investigated as a garbage bin to remove the proteins, lipids, and nucleic acids that are unwanted for cells. Over the past few years, exosomes have been known to convey biological molecules for various diagnostic and therapeutic purposes. Exosomes isolated from most of the cells are intrinsic carriers for endogenous protein molecules, suggesting that the exosome carrier system would be a suitable delivery approach for proteins or peptides [80]. Proteins such as enzymes, transmembrane proteins, and cytoskeletal proteins are reported to be delivered by exosomes. The exosome vesicles have been considered as a transporter of biomolecules, including lipids, proteins, and genetic material, owing to their ability to transfer their contents from mother cells to neighboring cells [8,81].

Naturally occurring exosomes contain membrane-associated protein ligands. Upon their biogenesis, these bioactive ligands cluster into microdomains on the exosomes, thereby providing a natural membrane environment for the biomacromolecules, which helps maintain stability and bioactivity, thereby contributing to elevating the efficiency of membrane protein therapeutics [82,83].

A number of proteins, such as heat shock proteins, annexins, and Rab family proteins, which are abundantly found in exosomes, are mainly involved in trafficking of exosomes and intercellular assembly, and inclusion of such proteins in exosomes may not be beneficial for drug delivery purpose. However, several other protein therapeutics may be exploited for exosome delivery purposes [35].

In the treatment of cancer, Survivin-T34A (dominant-negative mutant of the inhibitor of apoptosis protein Survivin) was successfully introduced into exosomes isolated from melanoma cell lines and played an important role in inducing apoptosis [84]. Kooijmans et al. anchored antiepidermal growth factor receptor nanobodies to the surfaces of exosome vesicles via glycosylphosphatidylinositol to improve the interactions between exosomes and epidermal growth factor receptor-expressing tumor cells [85].

Modified exosomes derived from the dendritic cells consisting of SAV (a protein that binds to biotin with high affinity) and LA (an exosome-tropic protein) were mixed with pH-sensitive GALA peptides to produce GALA-modified exosomes. These engineered exosomes are effective in controlling the intercellular transport and antigen presentation ability of tumor cells [86]. Similarly, alphagalactosylceramide or ovalbumin-loaded exosomes could potentially induce an adaptive immune response in the absence of triggering invariant natural killer T-cell anergy [87].

Tian et al. [88] conjugated exosomes on the surface of the c(RGDyK) peptide using bio-orthogonal chemistry for the treatment of ischemic stroke by targeting the lesion region of the ischemic brain. Further, these engineered exosomes were loaded with curcumin to suppress both the inflammatory response and cellular apoptosis in the targeted (lesion) region. The in vivo results for the cRGD–exosome delivery system showed encouraging therapeutic efficacy and targeting ability.

Some biomolecules are prevalent in all types of exosomes, including the generation of cytosolic proteins such as tubulin and actin, protein kinases, Annexin and Rab family proteins, tetraspanins (CD9, CD63, CD81), heat shock proteins (HSP 70, HSP 90), and various transmembrane proteins molecules [89]. Exosomes derived from antigen-presenting cells carry tetraspanin CD86 and major histocompatibility complex molecules I and II on their surfaces, enabling them to simulate CD8^+^ and CD4^+^ T cells. In addition, some molecules present at the exosome membrane may act as pathogen-associated molecular patterns, thereby contributing to the activation of immune cells [90]. Exosomes have the ability to increase and modulate immune responses, which could be one important strategy in the design of new vaccine formulations. The nanoscale exosomal vesicles are capable of stimulating innate and adaptive arms of the immune system, suggesting their potential to activate granulocytes or NK cells, and are also able to interact with CD8^+^, CD4^+^, and B cells in order to demonstrate antigen-specific immune responses [91].

In a previous study, Sandra et al. [92] investigated exosomes as potential vaccine adjuvants. Exosomes were isolated from lipopolysaccharide endotoxin (LPS)-stimulated human monocytic cell line (THP-1). The isolated exosomes were combined with a hepatitis B recombinant antigen (HBsAg) solution or suspension comprising HBsAg-loaded poly-ε-caprolactone–chitosan nanoparticles. The obtained results suggested that exosomes combined with HBsAg induced a humoral immune response similar to the control group, which is a HBsAg solution without exosomes. The findings of their study suggest that exosomes, when co-ingested with the antigen, could have potential applications to improve the protective immune response in vaccine development.

Liu et al. [93] investigated the utility of exosomes in neuronal recovery after ischemic stroke. Enkephalin-loaded exosomes containing a transferrin complex called enkephalin-tar-exo were developed to target the blood–brain barrier (BBB). In vivo delivery of the exosomal system showed that enkephalin-tar-exo crossed the BBB and decreased the levels of lactate dehydrogenase, p53, and caspase-3 when tested in rats using a transient middle cerebral artery occlusion–reperfusion model. In addition, the enkephalin-tar-exo system improved the brain neuron density and neurological score, indicating neurological recovery after stroke.

Barok et al. [94] examined the delivery of an antibody–drug conjugate (trastuzumab–emtansine) against HER2-positive cancer. Exosomes were isolated from several cell lines such as HER2+ (SKBR-3 and EFM-192A breast cancer), HER2 (MCF-7 breast cancer), and gastric cancer (SNU-216) via ultracentrifugation method followed by treatment with trastuzumab–emtansine. The results showed that antibody–drug-conjugated exosomes bound to HER2+ cancer cells with growth inhibition and activation of caspases-3, confirming the binding of trastuzumab–emtansine to HER2+ cancer cells.

Cho et al. [82] compared the efficacy between exosomes and the ferritin nanocage carrier in the delivery of signal regulatory protein α owing to the greater phagocytosis of tumor cells by macrophages, whereby the tumor growth inhibition induced by the exosome carrier was higher than that of nanocages. The abundance of proteins and lipids in the exosome vesicles offers a substantial advantage in providing an ideal microenvironment for membrane proteins regarding the activity and distribution in the membrane, reflecting their potential advantage over other delivery platforms.

Although exosome-associated proteins play an important role in triggering cellular responses and regulatory processes, the functional aspects of exosomes are complex in terms of their assembly, binding, fusion with targeted cells, and interactions with the extracellular matrix; for instance, paraformaldehyde-mediated crosslinking of proteins on the surfaces of exosomes decreased fusion of exosomes with parental cells by approximately 20%. Furthermore, exosomes that were treated through solubilization with octylglucoside and reconstructed by dialysis to remove the membrane proteins showed a decrease in their ability to fuse with target cells compared to untreated exosomes [95]. The fusion efficiency of exosomes with depleted proteins demonstrated comparable fusion efficiency to that of large unilamellar vesicles whose lipid composition was similar to that of naturally occurring exosomes, confirming the importance of exosome-associated proteins in fusion events [8]. Kim et al. [96] evaluated the potential of genetically modified exosomes to express a targeting ligand that can improve the exosome delivery to a target tissue and reduce the systemic toxicity. Briefly, the ability of cardiac-targeting peptide, a targeting ligand expressed from genetic modification of exosomes, to deliver tissues and heart cells in vitro and in vivo was investigated. Exosomes isolated from HEK293 cells via differential centrifugation were genetically modified by fusion of cardiac-targeting peptide (CTP)–Lamp2b on the membrane of the exosome (CTP–Exo), and exosomes expressing only Lamp2b (CTL–Exo) were used as a control. The in vitro study results showed that compared to CTL–Exo, a significant increase in the delivery of CTP–Exo was observed in H9C2 rat cardiomyocytes. In vivo studies showed that delivery of CTP–Exo was 15% more effective than CTL–Exo, confirming that genetic modification of exosomes with targeting peptides can be explored as a therapeutic tool for heart diseases owing to enhanced delivery and reduced systemic toxicity. Recently, for successful delivery across the BBB, exosomes loaded with the antioxidant protein catalase showed an improved disease state in Parkinson’s disease patients. As with many other actives, delivery of catalase across the BBB is a major hurdle, however loading of catalase into the exosome carrier system seems to be a promising approach for Parkinson’s disease therapy.

In a previous study, Haney et al. [97] incorporated catalase into exosomes using different methods, including incubation with or without the use of saponin permeabilization, freeze–thaw cycles, and extrusion and sonication procedures. Western blot analysis showed that sonication and extrusion techniques are the most effective in incorporating the catalase into exosomes. Further, lipophilic fluorescent dye was used to label the exosomes to confirm the delivery of catalase across the BBB. The exosomes labeled with fluorescent dye were incubated with a pheochromocytoma (PC12) cells. Confocal images confirmed the uptake of labeled exosomes in PC12 cells. When exosomal catalase was added, elimination of reactive oxygen species (ROS) was observed in in vitro-activated macrophages, suggesting successful delivery of exosome-loaded catalase and neutralization of ROS.

### 3.2. Exosomes in Gene Delivery

The triggering of RNA interference to induce gene silencing has been significantly intensified in biomedical applications. The RNA interference technique involves processing double-stranded RNAs into small interfering RNAs (siRNAs) via posttranscriptional, sequence-specific gene silencing. The siRNAs are used to selectively cleave target mRNA [98,99,100]. Several RNA-structure-related factors, including their negative charge, instability in the blood circulation owing to degradation by nucleases, immunogenicity, and need for a delivery vehicle, particularly when repeated dosing is needed to treat disease, limit the biomedical application of synthetic siRNAs [101,102]. The use of exosomes that can carry exogenous siRNAs to human cells can overcome these impediments. Exosomes as naturally occurring RNA carriers might be an effective source for the delivery of genetic material [103]. Owing to their natural ability to carry genetic material such as DNA and RNA to the targeted cells, exosomes have attracted increased interest in drug delivery involving genetic modification or alterations of gene expression in certain genetic therapies. Typically, small interfering RNAs (siRNAs) are used to disrupt the gene of interest. However, these siRNAs are prone to rapid degradation in the systemic circulation owing to their low stability. Exosomes as a carrier system help in both the protection and delivery of siRNAs to the targeted cells. Several studies have reported the utility of exosomes as therapeutic vehicles for the delivery of exogenous genetic material. There is a shortage of safe, efficient, target-specific therapeutic delivery vehicles for conveying genetic material. In the past, the ability of exosomes to transport endogenous mRNAs and microRNAs expressed by the exosome-producing cells to different cells in culture has demonstrated the concept of using exosomes for gene delivery [12].

Exosomes were used to deliver siRNA due to being non-immunogenic to the host and owing to their natural ability to deliver RNA from cell to cell. Wahlgreen et al. [104] used human exosomes to deliver siRNA to T cells and monocytes. Exosomes were isolated from various cell types, including lung cancer cells TB-177 and HeLa cells, then siRNA was loaded into exosomes via chemical transfection and electroporation methods. The results from confocal microscopy, flow cytometry, and Western and Northern blotting showed successful incorporation of siRNA into exosomes. Additionally, whether the delivered exosomes induced posttranscriptional gene-silencing in recipient cells was evaluated by performing immunoblotting analysis. The results showed that exosome-loaded siRNA caused a decrease in tagged siRNA against mitogen-activated protein kinase 1 (MAPK-1) expression, indicating successful gene slicing with downregulation of the specific gene, with these results suggesting the utility of exosomes as delivery vehicles in gene therapy. Similarly, Shtam et al. [105] evaluated the potential of exosomes to deliver siRNA to human cells by targeting RAD51, a gene protein that helps in repairing double-strand breaks of DNA. Exosomes isolated from HeLa cells using the centrifugation method were loaded with Alexa Fluor 488-labeled siRNA and then further co-cultured with HeLa and HT1080 cells. The results from the Western blot analysis showed that exosome-delivered siRNA downregulated the expression of RAD51 and RAD52. Skog et al. [106] reported that exosomes obtained from glioblastoma tumor cells could be used for diagnosis as they naturally contain miRNA in the exosomes. Using exosomes, the delivery success rate of siRNA was enhanced significantly. Moreover, exosomes released from the self-derived dendritic cells of mice had a more than 60% success rate in delivering siRNA specifically to the nervous system, which is much greater than that of the siRNA itself [107].

Exosomes isolated from different types of cells may have different compositions and functions. Exosomes released from endothelial cells are mostly associated with atherosclerosis and vascular inflammation. However, the ability of exosomes to deliver exogenous contents has not been explored greatly. In a study, Banizs et al. [108] investigated the potential of endothelial exosomes to deliver siRNA to endothelial cells. Exosomes isolated from endothelial cells using filtration and ultracentrifugation methods and then further loaded with siRNA using the electroporation technique were investigated for their capability to deliver siRNA to endothelial cells. Further, exosomes carrying siRNA were incubated in the presence of luciferase-expressing endothelial cells. The study results showed that endothelial-exosome-loaded siRNA expressed lower luciferase compared to the control group, suggesting the functionality of endothelial exosomes to deliver exogenous material to cells in vitro at the target site.

Typically, nucleic acid drugs can be loaded either endogenously or externally. The ability of nucleic acid drugs to exert a maximum therapeutic effect is important for vigorous exploration, gene expression, and maintenance of the physiological balance of the cells regulated by the delivery of specific functional siRNA to target cells. However, it is difficult for exogenous siRNA to penetrate the cell membrane and it can be easily degraded. One major challenge for the clinical application of gene therapy is the development of a suitable vehicle for diffuse delivery of genetic material to the brain. The ability of exosomes to load exogenous genetic cargo, the specificity imparted by the targeted exosomes, and their potential to systematically administer genetic material and invoke immune evasion are important properties for gene therapy applications [107,109,110].

Lydia et al. [107] loaded exogenous siRNA using dendritic-cell-derived exosomes through electroporation method, while engineering of dendritic cells was performed to express Lamp2b, an exosomal membrane protein, in order to achieve tissue-specific targeting. Further, the exosomes were injected with the targeting peptide RVG into mice intravenously to deliver GAPDH siRNA to neurons, oligodendrocytes, and microglia in the brain to show a specific gene knockdown. The study results showed that the exosome vesicles delivered GAPDH siRNA specifically to neurons in knockdown-related genes. The loading of exogenous linear DNA by exosomes using electroporation technique to explore the potential of delivering DNA to recipient cells in the clinical gene therapy was previously reported. However, the capacity of DNA and its loading efficiency depended on both the size of the DNA and the exosomes [111]. The delivery of nucleic acid drugs or genetic material via the exosomes involves fundamental treatment at the genetic level, which has gained greater attention in treating several diseases. Nevertheless, the usage methods, precise mechanism, and clinical effects in relation to safety considerations still need to be vigorously explored [86].

In recent years, studies have reported the successful delivery of siRNA to target cells. Faruqu et al. [112] loaded siRNA into exosomes for delivery to cancer cells. Exosomes isolated from HEK-293 cells by centrifugation method were fluorescently labeled and then loaded onto exosomes by electroporation method. The excess siRNA after loading into exosomes was removed using gel filtration. The results showed the efficient encapsulation of siRNA with promising exosome yield and successfully delivery into cancer cells.

Similarly, Limoni et al. [113] developed LAMP2B-DARPin-bearing exosomes to specifically bind to HER2/Neu, with subsequent delivery of the siRNA molecule against the TPD52 gene into SKBR3 cells. The results showed that exosome-loaded siRNA downregulated the gene expression of TPD52 by up to 70%, indicating the successful gene transfer to cancer cells by exosomes, providing an additional delivery system option for gene therapy. Zhang et al. [114] evaluated the efficiency of serum-derived exosomes in delivering siRNA via intratracheal instillation into lung macrophages to modulate lipopolysaccharide-induced lung inflammation. The results indicated that the exosome delivery system avoided the immune system, which is a major concern in delivering genetic material.

### 3.3. Exosomes in Delivery of Other Therapeutic Compounds

The vast majority of studies have investigated the exosome-based drug delivery systems for therapeutic transfer of interfering RNAs and other therapeutic cargo, while exosomes isolated from cancer cells for anticancer drug delivery systems have been explored for the potential loading of anticancer agents into exosomes. Exosomes are also promising delivery vehicles for small-molecule drugs owing to their small size, reduced toxicity, and bio-compatibility compared to nanoformulations such as liposomes and dendrimers. Drugs (particularly anticancer drugs) encapsulated in exosomes have demonstrated improved pharmacokinetic and pharmacodynamic properties and enhanced in vivo anticancer activity compared to free drugs [115]. Several studies have explored the use of exosomes in the in vitro and in vivo delivery of small-molecule drugs. Few studies have reported the superior therapeutic effects of exosome-loaded small-molecule therapeutics compared to free drugs or encapsulated carriers [116]. Schindler et al. [117] developed doxorubicin-loaded exosomes and studied their redistribution and rapid cellular uptake to the cytoplasm and nucleus. The doxorubicin exosomes demonstrated enhanced in vitro potency in multiple cell lines with increased cell uptake and redistribution when compared to free doxorubicin and its liposomal formulations. The exosome delivery system can provide benefits for both cell-based and nanotechnology-based drug delivery, allowing efficient transport of drugs, which can overcome the various biological barriers. Nevertheless, several factors need to be considered before using the exosomes for transporting drug molecules, such as the efficient drug loading of exosomes without causing significant changes in the exosomal membrane structure or content.

In cancer therapy, exosomes have the capability to interact with and accumulate in the target cancer cells. The exosomes found in Taxol are taken-up via endocytosis, owing to the presence of adhesion proteins, immunoglobulins, proteoglycans, tetraspanins, integrins, and lectins, meaning exosomes have superior uptake [118]. Furthermore, cellular membranes found in exosomes may fuse with endocytic membranes to deliver drugs, overcoming the Pgp-mediated efflux. Additionally, exosome-mediated cell-to-cell communication is important in the encounters between cancer cells and the immune system [119]. Parolini et al. [95] reported that the fusion of exosomes with target cells occurred more efficiently under acidic conditions, indicating the preferential uptake of exosomes by tumor cells, which have an acidic microenvironment, as compared to the surrounding healthy tissue. Since the exosome vesicles are small in size and native to the animals, they can be used to avoid phagocytosis and bypass the engulfment by lysosomes. Thus, most chemotherapeutic agents such as doxorubicin and paclitaxel are encapsulated into exosomes, showing potential for delivering into target cells [12].

Kim et al. [120] evaluated the feasibility of exosomes in delivering paclitaxel for multi-drug-resistant cancer. Various methods, including incubation at RT, electroporation, and mild sonication, were used to incorporate paclitaxel into exosomes. Of these methods, mild sonication provided the greatest loading capacity, which could be because a decrease in rigidity and the microviscosity of exosomal membranes upon sonication allowed the incorporation of paclitaxel into lipid bilayers of exosomes. The study results showed that paclitaxel-loaded exosomes greatly increased the cytotoxicity of drug-resistant MDCKMDR1 (Pgp+) cells, indicating the therapeutic potential of exosomes to treat drug-resistant cancers. The mechanisms likely to be responsible for the enhanced exosome-loaded anticancer activity included the efficient transport of paclitaxel into the target cancer cells, overcoming the Pgp-mediated drug efflux in the cells resistant to cancer, and the preferential accumulation of paclitaxel in targeted cancer cells.

In the past, curcumin encapsulated in exosomes has been shown to be more stable, being highly concentrated in blood and with a therapeutic effect [121]. Curcumin exhibits antioxidative and anti-inflammatory properties and could be a promising treatment for cerebral diseases. Accumulating evidence suggests that curcumin, when encapsulated in exosomes, induces exosome secretion and increases its solubility, stability, and therapeutic potential [122,123].

Kalani et al. [124] investigated the potential of exosomes when primed with curcumin in terms of endothelial cell dysfunction by studying their effects on oxidative stress, tight junction proteins, and endothelial cell layer permeability. The results demonstrated that oxidative stress in Hcy-treated cells was significantly decreased with exosome-loaded curcumin, suggesting its antioxidative potential. Likewise, curcumin-primed exosomes showed a beneficiary effect in amelioration of junction proteins and endothelial cell layer permeability by maintaining redox homeostasis, lowering the levels of MMP-9 and improving tight junctions, which eventually improved the endothelial cell permeability. In another study, exosomes were incorporated with curcumin to enhance the effectiveness of curcumin. Exosomes derived from a murine tumor cell line (EL-4) were mixed with curcumin and then subjected to sucrose gradient centrifugation. TSG101 and CD81 were used as exosomal protein markers to identify the exosome curcumin complex. Various in vitro and in vivo experiments were carried out to assess the anti-inflammatory activity of exosomal curcumin. The macrophages treated with exosome-loaded curcumin showed fewer inflammatory cytokines in vitro than those treated with curcumin alone, suggesting enhanced anti-inflammatory activity caused by the exosomal curcumin. In vivo, mice treated with exosomal curcumin demonstrated significant survival in a lipopolysaccharides-induced septic shock animal model compared to mice treated with curcumin alone [121].

Although exosomes have been proven to be useful carriers for delivering anticancer drugs, their in vivo delivery of anticancer drugs could be limited owing to their non-specific toxicity and off-target effects, which can be observed with the conventional delivery of chemo drugs. This necessitates the anchoring of tumor-targeted ligands such as peptides, antibodies, and aptamers with drugs loaded onto exosome surfaces to reduce the non-specific toxicity and allow tumor-specific drug delivery. Such ligand-equipped exosomes were reported to exhibit tumor growth suppression via a receptor-mediated endocytosis process, thereby overcoming endosome encapsulation and trapping [125].

Liu t al. [126] tested surface-modified exosomes with ligands to deliver doxorubicin. Exosomes isolated from the non-cancerous HEK293T cells were anchored with a ligand (lipophilic hyaluronic acid) to target CD44-overexpressing MCF7/ADR breast cancer cells. The ligand-anchored exosomes were shown to be elevated by doxorubicin in breast cancer cells, thereby decreasing the tumor mass by 89%. In addition to improving the properties of small-molecule drugs, exosomes are also used to transport drugs across the BBB. Owing to the problems associated with the permeability of small-molecule drugs across the BBB, most of the potent central nervous system drugs have not been successful in clinical trials because the majority of these drugs cannot cross the BBB. To compensate for these complications, exosomes as a body’s own cells are employed as delivery vehicles to tailor the drugs to cross the BBB, enhancing drug transport to the brain by decreasing mononuclear phagocyte system drug clearance [12,127].

**Table 2:** Examples of exosomes as drug delivery systems.

Cargo Type		Origin of Exosomes	Disease Type	Isolation or Purification Method	Drug Loading Method	Outcome	Reference
Proteins	Signal regulatory protein α	Human embryonic kidney293T cells	Cancer	Centrifugation	Transfection	Enhanced phagocytosis of tumor cells	[82]
	Survivin-T34A	Melanoma cell lines	Pancreatic cancer	Centrifugation	NA	Apoptotic death of cells	[128]
	Antiepidermal growth factor receptor	Mouse neuroblastoma	Epidermoid carcinoma	Ultrafiltration/size exclusion liquid chromatography	NA	Target specificity	[85]
	20S proteasome	Mesenchymal stem cells	Mouse myocardium	Tangential flow filtration	NA	Reduction in myocardial infraction	[129]
Genetic substances	miRNA	Glioblastoma cells	Glioblastoma tumor	Differential centrifugation	Transfection	Providing diagnostic information	[106]
	miRNA	Human cord blood endothelial colony-forming cells	Ischemic kidney injury	Centrifugation	Transfection	Protected kidney function and reduced kidney injury	[130]
	Spherical nucleic acids	PC-3 cells	Prostate cancer	Centrifugation	Naturally encased	3000-fold-enhanced knockdown of miR-21	[131]
	siRNA	Human embryonic kidneycells (HEK293)	Breast cancer	Sequential centrifugation	Electroporation	TPD52 gene expression was downregulated up to 70% compared with non-targeted exosomes	[113]
Small molecules	Paclitaxel	Prostate cancer cell lines (PC-3 and LNCaP)	Autologous prostate cancer	Differential centrifugation	Co-incubation	Enhanced drug cytotoxicity to cancer cells	[132]
	Doxorubicin	Immature mouse dendritic cells transfected with the vector-expressing iRGD-Lamp2b fusion proteins	Breast cancer	Centrifugation and ultrafiltration	Electroporation	Specific drug delivery to the tumor site andinhibited tumor growth	[116]
	Curcumin	Tumor cells (GL26-Luc, BV2, 3T3L1, 4T1, CT26, A20, and EL-4)	Brain tumor and autoimmune encephalitis	Sucrose gradient centrifugation	Direct mixing	Inhibited brain inflammation and delayed brain tumor growth	[121]
	Dopamine	Kunming mouse blood	Parkinson’s disease	Ultracentrifugation	Co-incubation	Enhanced therapeutic effect due to brain specific drug delivery	[133]

## 4. Exosomes Drug Loading Techniques

**Figure 3:**
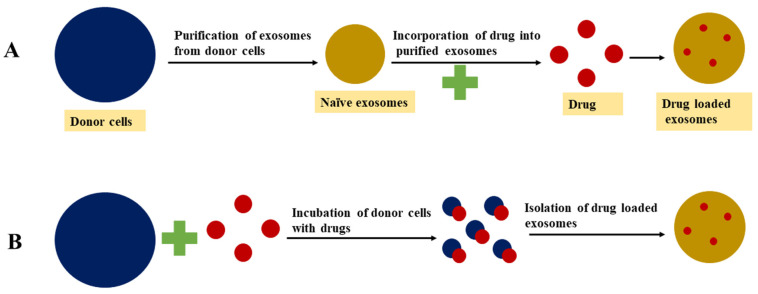
Exosomal drug loading approaches: (**A**) postloading approach; (**B**) preloading approach.

The lipid bilayer membrane of the exosome vesicle serves as a natural barrier to protect the degradation of cargo in the blood circulation. However, this lipid bilayer membrane, as well as the endogenous content of exosomes, makes drug loading into exosomes challenging [134]. Generally, active loading and passive methods can be used to sort the drug into the exosomes [135]. Active loading is also known as remote or postdrug loading, in which the drug is incubated with isolated exosomes. Passive drug loading, also termed as the preloading method, involves the secretion of drug-sorted exosomes from a pretreated donor or source cells. This method does not require the addition of drugs into the exosome vesicle. The active loading approach has been reported to be more effective in attaining a higher drug/vesicle ratio owing to its active pumping mechanisms. The postloading approach is more suitable for hydrophobic drugs than hydrophilic drugs [136,137]. Different approaches for drug loading into exosomes are presented in Figure 3, while the advantages and disadvantages of different exosome drug loading approaches are detailed in Table 3.

### 4.1. Passive Loading Approach

#### 4.1.1. Incubation of Drugs with Exosomes

This approach is also known as the passive drug loading method, in which both drug and exosomes are incubated together and the drug diffuses into exosomes along the concentration gradient. The drug loading efficiency using this method is directly related to the hydrophobicity of drug molecules because of the potential of hydrophobic drugs to interact with the lipid bilayer membrane of the vesicle [137]. In a study, Dongmel et al. incubated mouse lymphoma-derived exosomes with curcumin in PBS at 22 °C for 5 min, then the mixture was centrifuged based on a different sucrose gradient. The encapsulation of curcumin into exosomes enhanced the solubility, stability, and bioavailability compared to free curcumin [121]. Similarly, Vashisht et al. [138] reported that incubation of curcumin with exosomes resulted in a loading efficiency of 70.46%. Enzyme catalase was also encapsulated into exosomes via incubation in PBS at room temperature for 18 h. However, the low loading capacity is one of the main drawbacks associated with this method [97].

#### 4.1.2. Incubation of Drugs with Donor Cells

In this approach, the targeted exosome donor cells are treated with a drug molecule of interest and the pretreated cells then secrete drug-loaded exosomes [14]. The objective of this approach is for the donor cells to accumulate the bioactive or therapeutic compounds and secrete exosomes that can accommodate the therapeutic compounds. Owing to its untargeted nature, this approach may result in low exosome yield [137]. Pascucci et al. [139] treated and incubated SR4987 mesenchymal stromal cells with a low dose of paclitaxel for 24 h. Then, the cells were washed and reseeded in a new flask containing a fresh medium. After 48 h of culture, the exosomes loaded with paclitaxel were isolated and collected from the cell-conditioned medium. The pretreated donor cells may be exposed to mechanical or biological stimuli such as ultraviolet light, heat, or combination in order to release the drug-loaded exosomes [140,141].

### 4.2. Active Drug Loading Approaches

Active drug loading involves temporary disruption of the exosome membrane so that active cargo can easily diffuse into the vesicles. The integrity of the exosome membrane is then restored after the desired compounds are loaded into the exosomes. The various approaches used to disrupt the membranes of the exosomes include sonication, extrusion, and freeze–thaw cycles [135]. Compared to passive drug loading, the drug loading capacity of exosome vesicles increased up to 11 times using the active drug loading approach [136]. The main problem associated with this approach is the potential to damage targeting features and the native structure of exosomes during the membrane disruption process [135].

#### 4.2.1. Sonication

Exosomes derived from donor or target cells are mixed with a drug or protein of interest and then sonicated using a homogenizer probe. The mechanical shear force generated during sonication disturbs the exosome membrane’s integrity and allows bioactive compounds to diffuse into the exosome while deforming the membrane [137,142,143]. Kim et al. [120] reported that the microviscosity of the exosome membrane decreases significantly after sonication. However, the membrane-bound proteins or lipid contents of the exosome are not significantly affected by this membrane deformation process. It was found that the membrane integrity of the exosome can be restored within an hour when incubated at 37 °C. Moreover, in some cases, biphasic drug release is observed from the exosomes when the drugs are encapsulated inside of the exosomes and also attached to the outer membrane layer of the exosome vesicle. When the drug is attached to the exosome outer layer this results in burst release, whereas when the drug is encapsulated inside the exosomes this leads to slow release [120].

#### 4.2.2. Extrusion

Extrusion is a postloading method that employs a syringe-based lipid extruder for drug loading. Exosomes isolated from the donor cells are mixed with a targeted drug and then loaded into a syringe-based lipid extruder with a 100–400 nm porous membrane at a controlled temperature. During the extrusion, the drug is vigorously mixed with the disrupted exosome membrane [14]. Fuhrmann et al. [136] reported the benefits of the extrusion approach for the drug loading of exosomes. Exosomes derived from MDA-MB231 breast cancer cells were loaded with porphyrin using the extrusion method. Compared to the incubation method, extrusion loading resulted in a greater cytotoxic effect. Further, the extrusion method alters the zeta potential of original exosomes, while increasing the number of extrusions in the intensive extrusion process can contribute to effective drug loading due to the transformation of the vesicle constitution.

#### 4.2.3. Freeze–Thaw Cycles

Drug loading using the freeze–thaw approach involves incubation of exosomes with a targeted drug at room temperature for a given amount of time and rapid freezing at −80 °C or in liquid nitrogen. Then, the mixture is thawed at room temperature. For better drug encapsulation, freeze–thaw cycles are repeated for at least three cycles. This method has lower drug loading capacity compared to sonication or extrusion approaches. Additionally, this technique can promote the aggregation of exosomes, leading to broad size distribution of the drug-loaded exosomes [97,137,144].

#### 4.2.4. Electroporation

Electroporation utilizes an electric field, which facilitates the movement of drug molecules into the lumen of the exosomes by disturbing the phospholipid bilayer of the exosomes, thereby creating pores on it [145]. During electroporation, drug molecules diffuse through the pores formed on the exosome lipid bilayer membrane; meanwhile, the integrity of the membrane is recovered after the loading. This method is widely used for the loading of large molecules such as nucleotides (siRNA or miRNA) into exosomes [136]. The electroporation technique has low loading capacity owing to RNA aggregation and exosome instability issues. This technique can increase RNA loading into exosomes and enhance the loading of hydrophilic small molecules into exosomes [136].

#### 4.2.5. Incubation with Membrane Permeabilizers

Membrane permeabilizers and surfactants such as saponin can interact with the cholesterol in the cell membrane and form pores, which leads to exosomal membrane permeability. Compared to the incubation method, the membrane permeability method can enhance the loading efficiency of catalase into exosomes [146]. A previous study showed that an 11-fold increase in drug loading of hydrophilic molecules into exosomes was observed with saponin compared to the passive loading approach without saponin [136,137]. Using this method, the amount of saponin used for drug loading should be optimal and exosomes should be purified after incubation with saponin.

**Table 3:** Advantages and disadvantages of different exosome drug loading approaches [97,137,146,147,148,149,150,151,152,153].

Drug Loading Approach		Mechanism	Advantages	Disadvantages
Passive loading	Incubation of exosomes and free drugs.	Diffusion of cargo into a cell or exosomal membrane.	Simple operation. Does not compromise the membrane integrity.	Loading efficiency. Drugs may cause cytotoxicity to the donor cells.
	Incubation of the donor cells with free drugs.			
Active loading	Sonication	Creation of micropores for diffusion by mechanical shear force.	Higher loading capacity than the simple incubation method.	Sonication-induced membrane damage is a roadblock for large scale application. Influence on exosome integrity and cargo aggregation.
	Extrusion	Membrane recombination.	High cargo loading efficiency. Repeated extrusion provides a homogeneous blend of exosomes with cargoes.	Recombination of exosomal surface structure may compromise the immune-privileged status of exosomes, making exosomes visible to immune cells such as mononuclear phagocytes.
	Freeze–thaw cycles	Membrane fusion.	Simple and effective strategy to load various cargoes (drugs, proteins, and peptides) into exosomes directly.	Repeated freeze–thaw may cause protein degeneration and exosome aggregation. Drug loading efficiency is lower than sonication and extrusion methods.
	Electroporation	Creation of micropores for diffusion by the electric field.	High loading efficiency	The loading efficiency and aggregation of cargoes are major limitations.
	Incubation with membrane permeabilizers	Dissolves membrane molecules (cholesterol), create pores on the exosomal surface.	Higher loading capacity as compared with the simple incubation method	Saponin is hemolytically active in vivo, limiting the concentration (toxicity) of saponin used for drug loading. Extra purification process may be required to remove saponin.

## 5. Exosomes Administration Routes

To deliver the therapeutic agent or cargo-loaded exosomes to the target tissue or organ, several administration routes have been tested, including intravenous and intratumoral routes, which may lead to systemic distribution of exosomes. The route of administration can influence the tissue distribution of exosome-loaded drugs in vivo. The advantages of and diseases targeted by exosomes administered via different routes are presented in Table 4.

### 5.1. Intravenous Administration

Owing to its endogenous origin, exosome-based drug delivery should avoid hepatic clearance or removal by immune cells. When injected intravenously, exosome-loaded drugs can be delivered to several tissues, such as the brain, pancreas, and tumor tissues [154]. Similar to the other types of nanocarriers, intravenous injection of exosomes may favor extravasation and retention of the exosomes inside the tumor due to a lack of proper lymphatic drainage and the presence of leaky blood vessels in solid tumors [145]. Therefore, intravenous administration of exosomes is an appropriate delivery route, especially in malignancies. In a previous study, after intravenous injection, the pharmacokinetic profile of exosomes showed a half-life of around 2 minutes in systemic circulation, with minimal presence observed after 4 h [155]. The accumulation of exosomes in the liver and then in the lungs suggests that the clearance of exosomes from systemic circulation is comparable to that of other vesicular systems such as liposomes. In a previous study, exosomes derived from metastatic B16-F10 melanoma cells showed their distribution to the lungs, liver, bone marrow, and spleen when injected intravenously [156]. In a study by Morishita et al., exosomes derived from B16BL6 melanoma cells were radioactively labeled and then injected intravenously to measure the radioactivity by collecting blood and organs at different time points. After injection, at the 30 min time point, the injected exosome doses detected were 1% in the blood, 10% in the lungs, and 40% in the liver. These observations indicated that after intravenous injection, rapid clearance of exosomes from circulation was mainly driven by macrophages, which resulted in the accumulation of the injected exosome dose in the liver [157,158]. Although intravenous administration allows the exosomes to reach the target site, their short half-life index in circulation is one the major limitations of this route of administration [155]. However, additional modifications to the exosomes, including PEGylation of the exosome particles, can be effective in avoiding rapid clearance from the circulation after the intravenous injection by prolonging the half-life of exosomes in the circulation.

### 5.2. Intratumoral Injection

The intratumoral injection of exosomes loaded with a therapeutic agent is an appropriate administration route for cancer types, whereby the tumor is reachable without requiring major invasive manipulation. Previous studies have reported on reductions in tumor volume or dimensions after intratumoral injection of exosome-loaded therapeutic cargo to the tumor mass [159,160]. The advantage of this approach is that a direct injection of exosomes to tumor cells allows specific delivery of the therapeutics [145].

### 5.3. Intraperitoneal Route

Exosome administration via the intraperitoneal route allows the loading of larger exosome doses compared to other systemic administration routes. However, exosomes injected via this route rapidly dilute and expand to remote sites due to the vast area of the peritoneal cavity [161]. Few studies have reported the intraperitoneal injection approach for the delivery of exosomes.

In a previous study, Sun et al. [121] investigated the anti-inflammatory activity of curcumin when delivered by exosomes to the peritoneal cavity. To evaluate the potential of exosomal curcumin to increase the bioavailability of curcumin, both free curcumin and exosomal curcumin were administered intraperitoneally at doses of 100 mg/kg. Intraperitoneal administration of curcumin resulted in five- to ten-fold higher accumulation of curcumin in peripheral blood than with free curcumin. At 12 h after injection, a group of mice injected with exosomal curcumin showed detectable curcumin in their plasma, whereas no detectable curcumin was observed in the plasma of mice treated with curcumin alone.

### 5.4. Oral Administration

Although oral administration is convenient, easy, and facilitates patient compliance to treatment, exosome delivery using this route involves several obstacles, such as enzymatic activity and changes in the pH and intestinal barrier along the gastrointestinal tract. The existence of severe acid–base changes and the characteristics of the intestinal microflora are issues that need to be overcome in order for the exosomes to reach the target tissue of interest. Oral administration of exosomes is more successful delivery to intestinal luminal epithelial surfaces than in non-gastrointestinal tissues [161]. Agrawal et al. [162] utilized bovine-milk-derived exosomes for oral delivery of paclitaxel for improved efficacy and reduced toxicity. Paclitaxel-loaded exosomes showed excellent stability in the presence of simulated gastrointestinal fluids. Following oral delivery, significant tumor growth inhibition was observed against human lung tumor xenografts in nude mice. Compared to intravenous administration, oral delivery of paclitaxel-loaded exosomes resulted in reduced systemic toxicity and inflammation. Aquil et al. [163] reported on the oral delivery of curcumin using milk-derived exosomes. Oral administration of exosomal curcumin resulted in significant inhibition of a cervical tumor xenograft. Additionally, enhanced antiproliferative activity against various cancer cell lines was observed after oral delivery of exosomal curcumin compared to free curcumin. However, depending on the administered dose, higher levels of curcumin were found in the lungs, liver, and brain in a dose-dependent manner.

### 5.5. Intranasal Administration

The intranasal administration route is more effective, particularly in circumventing the challenges involved in delivering drugs across the blood–brain barrier (BBB). The intranasal route diminishes the exosome loss by avoiding the intestinal and hepatic metabolism, thereby retaining the exosome vesicles in the brain tissue [145]. Studies have reported that intranasal administration of exosomes loaded with curcumin and cucurbitacin resulted in rapidly delivery to the mouse brain. Using this route, cucurbitacin-loaded exosomes increased the rate of tumor apoptosis and exhibited a reduction in disease progression in mice models. Curcumin-loaded exosomes showed a significant reduction in the microglial cell number [122]. The intranasal delivery of exosomes has also been successfully used to transport therapeutic cargo to inhibit inflammation and cancer of the brain [122]. Furthermore, the exosomes used in intranasal administration showed promising results and were reported to be more effective in mouse models for Parkinson’s disease therapy [97].

**Table 4:** Advantages and targeted diseases of exosomes administered via different routes [145,164,165,166,167,168].

Routes of Administration	Targeted Disease	Advantages
Intravenous	Stroke, Parkinson’s disease, traumatic brain injury, acute kidney injury, antitumor therapies (prostate and breast cancer).	Most common route for systemic administration of exosomes.
Intraperitoneal	Bronchopulmonary dysplasia Autoimmune type 1 diabetes	Allows the loading of larger EV doses.
Oral	Facilitates resolution of colitis, arthritis	Convenient administration route for patients.
Intranasal	Brain parenchyma Brain cancer Encephalitis (inflammation of the brain), Parkinson’s disease therapy	Suitable for EV delivery into the brain, surpassing the blood brain barrier.
Intratumoral	Glioblastoma multiforme antitumor therapies	More effective strategy for antitumor therapies due to higher EV retention in tumors.

## 6. Characterization Techniques

After isolation of the exosome, the exosome samples should be characterized thoroughly using a set of combination methods to validate the isolation method. One major challenge in exosome biology is the accuracy of the methods in measuring the quantity and purity of exosomes. The characterization methods used for measuring the exosome purity are categorized into marker-based, biophysical, and imaging-based methods. The advantages and limitations of exosome characterization techniques are presented in Table 5.

### 6.1. Imaging

An imaging tool is a qualitative technique used to determine the morphology of exosomes. Owing to the size of exosome vesicles, microscopic techniques may not show sufficient resolution to image exosomes. Atomic force microscopy (AFM), scanning electron microscopy (SEM), and transmission electron microscopy (TEM) are commonly used imaging techniques that can allow high-resolution exosome imaging.

#### 6.1.1. AFM

The AFM imaging technique detects and measures the force between the probing tip and sample surface in order to produce a topological map of the sample. AFM uses surface scanning with a sharp tip on the cantilever to scan over a sample surface. When the tip approaches the sample surface, the attractive force between the surface and the tip makes the cantilever deflect towards the surface and provides sub-nanometer, high-resolution imaging at less than 1 nm [169]. Briefly, a sample containing an exosome vesicle is placed on a mica substrate, dried at room temperature, then the dried samples are subsequently washed and allowed to dry in the presence of liquid nitrogen. Here, the dried sample can be viewed under AFM using a silicon probe and analyzed with software [170]. The AFM technique requires minimal sample preparation and can be used to measure the exosome vesicle in native conditions with a non-destructive mode of operation. This technique provides useful information related to the morphology, biomechanics, and biomolecular characteristics of the exosomes. Several studies have reported the effective use of AFM in characterizing the membrane composition, mechanical properties, morphologies, and sizes of various types of cell-derived exosomes [171].

#### 6.1.2. TEM

TEM is widely used to characterize the existence of exosomes in solution and to study the structure, size, and morphology in order to assess the quality of exosomes [172]. TEM uses an accelerated electronic beam with a smaller wavelength than that of light to determine the structure and morphology characteristics. The principle involved is the generation of the image as a beam of electrons that passes through a sample, whereby a secondary electron is generated [171]. Briefly, a suspension of exosome vesicles is fixed with paraformaldehyde (2% *w*/*v*), then deposited onto formvar–carbon-coated grids and incubated for 20 min. The carbon-coated grids are then washed with PBS, incubated with glutaraldehyde, a crosslinking agent, and washed with water. Finally, the exosome vesicles are stained with uranyl acetate solution (2% *w*/*v*) and then air-dried [173]. During TEM sample preparation, the morphology of exosomes can be affected due to the involvement of multiple steps, and often electron beams may induce changes in the morphology of exosomes. Hence, the TEM technique can be upgraded (Cryo-TEM) to eliminate the effects related to sample preparation by utilizing a different protocol [65].

#### 6.1.3. SEM

In the SEM technique, accelerated electrons carry a significant amount of kinetic energy, which is dissipated as different signals produced by the interactions between electron samples while the incident electrons are decelerated in the solid sample. Briefly, exosome samples are fixed on a carbon-coated or copper grid with glutaraldehyde and dehydrated with ethanol. The grids are then air-dried and sputter-coated with gold at a thickness range of 2 to 10 nm, then the samples are analyzed using SEM. In one study, it was reported that images of exosomes were round and bulging in appearance when observed using SEM [14,174,175].

### 6.2. Dynamic Light Scattering (DLS)

DLS, also known as photon correlation spectroscopy, works on the principle of time-dependent fluctuations in scattering intensity caused by Brownian movements of the particles within a sample [176]. DLS is the most suitable technique for measuring monodisperse suspensions (one type of particle in a suspension). When large vesicles are present in the suspension, even at low quantities, which may be problematic for the detection of small particles [177], this technique can be used to determine the vesicle size of the exosomes, however it does not provide information about the source or biochemical data for the exosomes [178]. However, the DLS method has some limitations in the characterization of exosomes, as follows: this technique requires a high sample (particle) concentration, which may be challenging to prepare for exosomes; the presence of larger particles in the sample results mask the exosome population due to the influence of larger particles on the intensity distribution; this technique is unable to accurately determine the particle concentration; the low scattering properties of the exosomes can make measurements inaccurate [174].

### 6.3. Flow Cytometry

Flow cytometry is a technique that passes individual cells through a laser beam at a specific wavelength and detects the emitted fluorescence or scattered light. This technique allows the measurement of the size and structure of the exosome, is used to characterize the exosomal surface proteins, and has the potential to determine the cellular origin of a single exosome vesicle [171,179]. Using this technique, Melo et al. [180] reported that exosomes isolated from non-tumorigenic cells carry less glypican-1 when compared to exosomes obtained from pancreatic cancer cells. Conventional flow cytometry has a detection limit of 200–500 nm, which limits its use in measuring free exosomes. When the flow cytometry technique is optimized to detect small particles it can then be used to detect the exosomes, however the low detection limit (approximately 100 nm) indicates the insufficient sensitivity of this technique. However, the sensitivity of the instrument in detecting exosomes can be improved by decreasing the wavelength of the laser beam to 405 nm [179,181].

### 6.4. Nanoparticle Tracking Analysis (NTA)

The NTA technique relies on the same basic principles as DLS. However, in NTA, a microscope is used to capture the individual particles in Brownian motion. NTA can measure the exosome concentration and size distribution in the range of 10 nm to 2µm. It allows one to measure the path of exosomal movement by tracking individual particles through image analysis, then this movement can be correlated to estimate the hydrodynamic diameters. As an individual particle is imaged in different regions, this technique can detect particles of different sizes in the sample. In addition, NTA can also have fluorescence capability, meaning is can be used to detect the antigen present on the exosome by applying fluorescently labeled antibodies [182]. The pre- and postprocessing settings used for NTA, such as changes to the camera sensitivity and detection threshold for a particle, can influence the results [183]. The sample preparation and correct dilution factor are two important parameters for the success of NTA. The ability of NTA to detect exosomes with diameters as low as 30 nm, its speed, and the ease of sample preparation and recovery in their native form after the measurements makes this technique more attractive [184].

### 6.5. Tunable Resistance Pulse Sensing (TRPS)

TRPS is a biophysical technique that involves the passing of single particles through nanoscale pores. The duration and frequency of resistance pulses are detected when the particles pass through the pores. This information is used to determine the concentration, size, and zeta potential. TRPS can determine colloidal particles with diameters ranging from 50 nm up to the size of cells, which is crucial when investigating cellular functions and uptake. When compared to the NTA, the size distribution and particle concentration of exosomes more closely resemble the true distribution as measured by TRPS. While making measurements using TRPS, frequent pore blocking by particles and susceptibility to system suitability issues have been reported [181,185].

### 6.6. Protein Characterization

Characterization methods based on proteins or markers can be used to confirm that the isolated exosomes contain low levels of potential contaminants and do not have high levels of exosome markers. The total exosome protein content can be quantified by measuring the total protein assay. However, discriminating the exosomal proteins from non-exosomal proteins is a challenge when using this method, which is mainly due to the co-isolation of exosomes with non-exosomal proteins during the isolation process. Owing to the endosomal origin, exosomes isolated from most of the sources will contain proteins (membrane or intraluminal proteins) involved in the endosomal formation. The characterization of protein markers can be performed using Western blot or ELISA techniques [11,186].

#### 6.6.1. ELISA

ELISA is plate-based assay technique used for the detection and quantification of the protein content of the exosomes. Its low sensitivity and the need for a large sample volume are some of the disadvantages of this technique. The ELISA technique can be used to measure exosome counts with accuracy [187].

#### 6.6.2. Western Blotting

The Western blotting technique is most commonly used to detect the presence of target proteins associated with exosomes. Briefly, purified exosome samples are treated with buffered lysis solution containing denaturants or protease inhibitors, then dodecyl sulfate–polyacrylamide gel electrophoresis is used to separate the protein lysates before being transferred onto a membrane for immunoblotting of specific protein targets. This technique is useful in determining the sizes of different proteins. However, the lengthy preparation and processing times involved are the major drawbacks of this method. Both ELISA and Western blotting techniques have similar limits of detection, however ELISA can be scaled up for high-throughput measurements [188,189].

**Table 5:** Methods, advantages, and limitations of exosome characterization techniques [172,179,188,190,191,192,193,194,195].

Identification or Quantification Methods	Purpose	Advantages	Limitations
Dynamic light scattering	Exosomes size distribution.	The lower measurement limit is 10 nm, suitable for the determination of monodisperse systems. Sample preservation for downstream analysis and requires no sample preparation.	Difficult to distinguish contaminated proteins with exosomes, not suitable for measuring complex exosome samples with large size ranges. Inaccurate with polydispersed and heterogeneous samples.
Nanoparticle tracking analysis Technology	Measurement of size and concentration of exosomes.	Higher resolution than flow cytometer, exosomes can be observed in real time with faster detection speed.	Detection threshold and camera levels will affect the quantification of exosomes.
Atomic force microscopy	Detection of exosomal morphology.	Require small sample amount, no sample fixation or staining.	Sample dehydration on mica surfaces may lead to modifications of the size and morphology of exosomes.
SEM and TEM	Detection of exosomal morphology.	SEM can be used to directly observe the surface structure, whereas TEM can be used to observe the internal structure of exosomes and provide information about particle size distribution.	SEM resolution is lower than TEM, high requirements in terms of sample preparation make TEM not suitable for rapid measurement of a large number of samples.
Flow cytometry	Detection of biomarkers of exosomes.	Qualitative and quantitative characterization of exosomes.	Detection limit is 400 nm, identification of multiple vesicles as a single event is possible, the particle size of exosomes cannot be measured, detection of proteins or antibody aggregates limits its application.
ELISA	Exosome protein quantification.	Suitable for high-throughput analysis and rapid detection with high specificity, can be used to analyze the marker proteins quantitatively and qualitatively.	Time-consuming, possible detection of non exosomal marker proteins, complicated operation with less repeatability.
Western blot	Exosome marker protein quantification.	Easy to analyze exosomes from cell culture media, the classic method for qualitative and quantitative analysis of marker proteins.	The detection of exosomal marker proteins varies depending on the type of parental cell, meaning this technique not suitable for the detection of exosomal marker proteins in biological fluid. Provides non-specific information on exosome concentration and size or distribution.

## 7. Manufacturing of Exosomes

**Figure 4:**
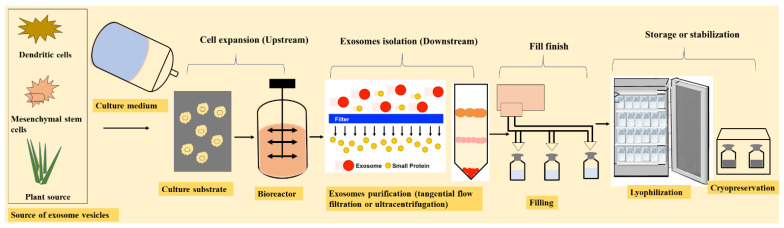
Schematic representation of the manufacturing scheme (upstream and downstream processing) for exosome-based therapeutics.

Exosomes are a novel form of biotherapeutics, and their manufacturing (production and purification) is similar to biologic production in terms of the cell culture and purification process. Exosome production involves a culture of the parent cell line, harvesting from the conditioned medium, and separation or purification from the process-related contaminants as extracellular vesicles. The exosome manufacturing process is divided into two stages: upstream and downstream. The upstream and downstream process workflow for the generation of exosome-based therapeutics is shown in Figure 4.

### 7.1. Upstream Processing

In exosome manufacturing, the cell source and cell culture media are the most important starting components. The cell culture media mainly employed in the production of exosomes include mesenchymal stem cells, dendritic cells, HEK293 cells, and 293T cells. The exosomes secreted by mesenchymal stem or stromal cells (MSCs) are well studied for the treatment of a range of therapeutic conditions. The MSCs can be isolated from different tissues, including adipose, bone marrow, and umbilical cord tissues [196]. In the early stages of product development, it is necessary to screen multiple cell types to identify the optimal cell source for a specific therapeutic efficacy and indication.

The cell culture medium is used to isolate cells and support the expansion of the parent cell line. Fetal bovine serum (FBS) and human platelet lysate (hPL) are standard cell culture media components. The cultivation medium is classified into animal-free or animal-derived components as dissociation enzymes are utilized in the manufacturing process [197].

The presence of a significant amount of endogenous exosomes in both FBS and hPL is an important issue for these media, which can contaminate the secreted exosome product [198]. In such cases, a serum-free medium (SFM) is an alternative option to allow exosomes of the desired quality. Importantly, the choice of medium should be considered early in development, particularly for media involving serum deprivation, because this can affect the protein content and function of the cell line [199]. The cell cultivation technique involves static (flasks) and dynamic (bioreactors) systems. In a previous study, the static flask system used was a stand tissue culture flask and a CellBIND^®^ surface with a negative surface charge, which was pretreated with an oxygen-containing functional group [200,201]. Bioreactors are dynamic monitoring systems used for large-scale production. The type of bioreactor used can affect the exosome yield by affecting the cell density, secretion, and reuptake by the cells. For exosome production, T-flask or hollow fiber bioreactors have been commonly used [202]. Owing to the size of exosomes (60–200 nm), a hollow fiber bioreactor with a molecular weight cutoff membrane is often used for harvest conditions. Such bioreactor systems facilitate a dynamic environment for cell cultivation and a continuous medium collection system, which is beneficial for downstream purification [200]. Microenvironmental controls in the bioreactor, including control of the oxygen, carbon dioxide, temperature, and homogeneous nutrient transport, can affect the quality of exosomes.

### 7.2. Downstream Processing

Typically, the downstream processing consists of filtration to remove the cell debris, concentrate the cell culture condition medium, and isolate exosomes from the concentrated condition medium. After cell harvest, a variety of methods can be used to purify exosomes, including ultracentrifugation, microfiltration, size exclusion chromatography, and immunoaffinity.

The current purification methodologies are based on separating exosome vesicles from cells, media, and proteins depending on the vesicle density, size, and surface markers. Each separation or purification method isolates a slightly different exosome population; hence, there is no standardized method for separation of exosomes. However, the choice of downstream processing depends on the target product profile and complexity of the starting material from the upstream operation [203].

Historically, ultracentrifugation is a commonly used method for efficient purification. Ultracentrifugation involves two main variations. The first variation employs a combination of centrifugal forces (3000–10,000× *g*) to reduce contamination associated with cell debris or fragments, followed by a centrifugal force measuring 10,000–20,000× *g* for organelles and non-exosomal vesicles, then a centrifugal force measuring 100,000–120,000× *g* before producing a final pellet of exosomes. The second variation discriminates the exosomes from other vesicles through flotation using commercially available reagents, such as iodixanol or density gradients obtained from deuterium oxide–sucrose cushions [70,204]. Exosome vesicle aggregation or destruction may occur after harvesting the exosomes via ultracentrifugation because of elevated shear associated with the ultracentrifugation process, which can break down the exosomes and release or leach the proteins from the exosomes [205].

In recent years, the TFF technique has been used to concentrate exosomes from cell culture media based on their size distribution. Tangential flow filtration can facilitate the buffer exchange and product washing, making this process attractive as a primary recovery method. Compared to ultracentrifugation, exosomes obtained from tangential flow filtration have shown greater immunomodulatory potency, which is similar to that of the parental cells. Moreover, the exosomes obtained from TFF show more soluble factors in the exosomes, including cytokines, proteins, DNA, RNA, and lipids [205].

In addition, hollow fiber ultrafiltration coupled with microfiltration enables the removal of large particles and cell-culture-derived proteins while retaining the structural and functional integrity of the exosomes [204,206,207,208]. Immunoaffinity is the most promising method for exosome purification; however, this method is the least reported in the literature as compared to the recovery of exosomes from a human colon cancer cell line (LIM1863) using different purification methods [68,209]. Study results have shown that exosomes captured via immunoaffinity demonstrate superior expression levels of known exosomal markers compared to other methods, such as ultracentrifugation and differential centrifugation. Further, immunoaffinity isolation enables identification of the ESCRT-III component VPS32C/CHMP4C and the SNARE synaptobrevin 2 (VAMP2) molecules in the exosomes, indicating the potential of the immunoaffinity method for exosome characterization and isolation.

Size exclusion chromatography (SEC) is a suitable purification method for removing protein aggregates and lipoprotein particles. SEC provides a significant decrease in the total particle number (by 30–70%). Although upscaling is possible with SEC, this method may not be suitable for the initial volume reduction required for cell-derived exosomes. However, compared to ultracentrifugation, a 100-fold decrease in ferritin (major protein complex contaminant) concentration has been reported in SEC-purified exosomes [202,210].

### 7.3. Fill Finish

After the purification of exosomes, the exosome product must be stored in a suitable container closure system under cryopreservation in a storage buffer that maintains the exosomes’ vesicle stability. Cryopreservation (−80 °C) with cryoprotectants is frequently used to reduce osmotic damage and enhance the stability of proteins and cells during freezing [211].

## 8. Stability and Stabilization Methods

Although exosomes have sparked interest in a variety of applications as cell-derived biotherapeutics and drug delivery vehicles, preservation and storage remain major challenges, which are yet to be studied extensively and must be addressed to enable their use for delivery systems. Previous studies suggest that freezing (−80 °C) is a promising mode of storage for exosomes. However, the effects of storage may vary with the source of isolation. The lack of knowledge about appropriate storage and stabilization conditions for exosomes and the limited understanding of storage-mediated effects may hinder the widespread clinical applications of exosomes for drug delivery [212].

In most cases, depending on the source of isolation, exosomes are not stable even under long-term storage conditions at −80 °C. Compared to freshly isolated exosomes, a change in the exosomes’ morphology was observed when stored at −80 °C for 4 days. Briefly, freshly isolated bronchoalveolar lavage fluid (BALF) exosomes have a characteristic morphology and mean diameter. The diameter of BALF exosomes increases by 10% and 25% when stored at +4 °C and −80 °C, respectively, due to multilamellar structure formation. Moreover, exosomes stored at different temperatures can show leakage of distinct protein groups or dissociation of pre-exosomal proteins loosely bound to exosomes. These observations indicate that storage conditions destabilize the morphological features, surface properties, and protein content of BALF exosomes [213]. Sokolova et al. [174] examined the stability of exosomes when stored at −20 °C, 4 °C, and 37 °C. The size of the exosomes was decreased under storage conditions of 4 °C and 37 °C, indicating an exosomal structural change or degradation. Further, multiple ultracentrifugation, freezing, and thawing did not affect the size of the exosomes. Thus, the size and integrity of the exosomes greatly depend on the storage conditions.

Similarly, Wu et al. [172] characterized the quality of RNA contained within the exosomes when stored under different storage conditions. A long-term storage period (2 years) also degraded the exosomal RNA, even when samples were frozen at −80 °C. Moreover, a decrease in the biological activity of exosomes was observed during storage at −80 °C for 28 days, highlighting the potential changes during the storage of exosomes and emphasizing the storage conditions that need to be considered while developing exosomes for clinical use [214].

In order to obtain viable and effective exosomes for drug delivery applications, the preservation technique should protect the contents of exosomes during both preparation and storage. Various approaches to preserving the physical characteristics of exosomes in solution and solid state are crucial to maintaining biological activity and reproducibility in downstream processing. During standard isolation procedures, problems related to particle aggregation during high-speed centrifugation of exosomes, interactions of highly enriched extracellular vesicle suspensions during storage, and damage caused by freezing are recurrent problems linked to exosome delivery systems [42,215]. The clinical therapeutic potential of exosomes as delivery vehicle can be widened by developing a suitable preservation method. Although a freezing temperature of −80 °C is generally used for the storage of exosomes, this temperature may not be ideal for transportation and handling. Therefore, other methods are required to improve the storage stability of exosomes. Cryopreservation and lyophilization or freeze-drying are effective storage methods that can be used to preserve exosomes [216]. Cryopreservation includes freezing of exosomes, thawing, and possible refreezing after usage, which preserves the exosome functions by lowering the temperature below that required for the biochemical reaction. However, cryopreservation may be associated with stress caused to the exosome vesicles. This stress is due to intracellular ice formation and osmotic imbalance within the freezing procedure [217].

In a previous study, the exosomal protein, RNA, and exosome marker were greatly reduced when stored at room temperature for 10 days compared to similar exosomes stored at −70 and 4 °C. Flow cytometry results revealed that when incubated at room temperature for 10 days, the exosome population became more dispersed than freshly isolated exosomes or exosomes incubated at −70 °C [218]. In a previous study, it was concluded that temperatures above −20 °C were not suitable for the preservation of intact exosomes. Some of the study results showed that exosomal RNA and the stability of exosome vesicles isolated from plasma were not affected by repeated freezing and thawing [174]. Other studies have stated that the exosome vesicle structure can be sensitive to repeated freezing and thawing [172]. Thus, although the cryopreservation technique is favorable in terms of its simplicity and availability, temperatures above −20 °C are not suitable for exosome preservation and repeated freezing and thawing may affect the exosomes. Hence, dehydration or drying by lyophilization appears to be a suitable technique for the preservation of exosomes [217].

Lyophilization is a preservation method for effective and long-term storage of exosomal formulations, which can enable storage at room temperature. Lyophilization is the preferred method over freezing, owing to the improved stability of exosome contents (proteins or RNA) resulting from the removal of freezable water associated with the exosomal content [219].

The lyophilization process consists of freezing (solidification) and primary drying (sublimation), followed by secondary drying (desorption) [220]. Sublimation is the basic principle involved in the lyophilization preservation process. Similar to cryopreservation, stresses associated with the freezing and dehydration steps of the lyophilization process may lead to destructive effects on the structural content of the exosomes. Thus, the addition of cryoprotectants or lyoprotectants in the formulation can protect the exosome vesicles and their cargoes [217].

Lyophilization can prevent the hydrolysis of phospholipids, thereby decreasing the physical degradation of vesicles during their shelf life. In addition, it may also help in the stabilization of the active components incorporated in the exosomes. Typically, sugars, especially trehalose, have been used as cryo- or lyoprotectants during lyophilization, effectively preventing leakage and protecting the membrane integrity due to their higher Tg values, making them suitable stabilizing agents during the lyophilization of exosomes. Trehalose is a non-reducing homodisaccharide composed of two glucose units linked by an α-1,1-glycosidic linkage. It has been reported that the addition of trehalose to exosomes narrows the particle size distribution and enhances the number of individual particles per microgram of protein. Moreover, trehalose can minimize the aggregation of vesicles caused by freeze–thaw cycles and maintain the particle characteristics of the lyophilized exosomes. Moreover, in in vitro electroporation experiments, trehalose can minimize the vesicle fusion and loss of exosomes during lyophilization [221,222].

Generally, cryoprotectants are divided into intracellular agents or penetrating cryoprotectants and extracellular compounds (non-penetrating cryoprotectants). Intracellular agents (dimethyl sulphoxide, glycerol, and ethylene glycol) prevent the formation of ice crystals by penetrating the cells, subsequently avoiding membrane rupture. Extracellular compounds such as sucrose, trehalose, and other sugars that do not penetrate the cell membrane act via different mechanisms [217,223]. Trehalose primarily acts through water replacement and vitrification mechanisms during exosome lyophilization. The water replacement mechanism involves the replacement of water molecules by formation of a stable hydrogen bond between sugars (trehalose) and exosome lipids at the bilayer surface, without affecting the lipid bilayer structure upon removal of water. Moreover, sugars maintain the spacing between lipid head groups and reduce the van der Waals interactions between acyl chains of the phospholipids to retain sealed membrane structures during lyophilization [220,224]. Vitrification represents the kinetic stabilization related to the Tg of the glassy matrix, which is based on the immobilization of molecules (proteins or lipids) by the glassy matrix of the stabilizer (trehalose) upon removal of water. Thus, the glassy matrix, having low mobility and high viscosity, prevents the aggregation or fusion and protects the lipid bilayers or protein molecules from damage from the ice crystals. The presence of trehalose also inhibits the conformational changes observed from the lipid phase transition [176,225].

Charoenviriyakul et al. [216] developed lyophilized exosomes for preservation at room temperature and compared their properties with those of the exosomes stored at −80 °C. The presence of a cryoprotectant (trehalose) during lyophilization greatly prevented the aggregation of B16BL6-melanoma-derived exosomes. In contrast, lyophilization without trehalose resulted in the aggregation of exosomes owing to stresses generated during this process. During lyophilization, proteins and RNA in exosomes were protected in the presence of trehalose. Additionally, exosomes after the lyophilization retained the activity and immunomodulatory of Gaussia luciferase and CpG DNA, respectively, for 4 weeks when stored at 25 °C, suggesting that the lyophilization of exosomes with trehalose is an effective storage method that can be used to preserve exosomes for various applications.

Bosch et al. [221] investigated the potential benefits of trehalose in phosphate-buffered saline (PBS) to maintain the stability and functionality of beta cell exosome-like vesicles during isolation and storage. The addition of trehalose significantly decreases the aggregation of exosome vesicles when compared to PBS alone and preserves the integrity by reducing the exosome vesicle loss during isolation and storage, helping in improving the stability of exosome vesicles for large-scale applications. Typically, trehalose provides a physical shield effect through different mechanisms, including vitrification, preferential exclusion, or water replacement theories [176]. The exosomes’ lipid bilayer may be damaged by the ice crystals that are formed during the freezing step of the lyophilization process. Additionally, vesicle fusion or phase separation may occur during dehydration and rehydration, respectively [225,226]. In a previous study, Akers et al. [227] investigated the impacts of storage conditions on cerebrospinal-fluid-derived exosomes. They found that lyophilization in the absence of trehalose resulted in a reduction in the number of exosome particles, which was likely due to aggregation, indicating that lyophilization without a cryoprotectant, particularly trehalose, damages the exosome vesicles. Therefore, it is necessary to investigate the appropriate preservation technique and optimal amount of cryo- or lyoprotectant required to preserve and extend the biological effect of exosomes or exosome-loaded cargoes to facilitate their clinical application.

## 9. Scalability Challenges

The endeavor to bring therapeutic exosomes into industrial-scale production and clinical use will require scalable cell culture conditions and methods related to the isolation and purification of exosomes. Further, storage conditions that maintain the functionality of exosomes must be utilized, and the entire production environment should adhere to current Good Manufacturing Practices (cGMP). The scaling-up of cell-culture-conditioned media needs to be assessed for exosome products based on the cell culture method used (adherent or suspension type cell culture), quantity of exosomes produced by the suitable cells, and the amount of exosomes required for therapeutic administration [1]. Studies have reported that MSC and cardiac progenitor cells can be obtained from bioreactor and HyperStack systems, respectively, in clinically relevant amounts. The results indicate that neither cells nor exosome vesicles change the phenotype while scaling-up the cell culture [200,228]. Cellular changes may occur while transitioning from a bench-scale cell culture to scalable cell culture platforms, which often alter the composition and function of the exosomes. The use of large-scale stem cell cultures might be a rate-limiting step in producing effective and stable products owing to high development costs and uncertainty in meeting regulatory and marketing requirements. Similarly, opportunities for delivering large quantities of cell-culture-conditioned medium with which to undertake the scaling-up of exosome production are limited [204,229].

When scaling up the cell culture, maximization of the surface area in stirred bioreactors or hollow fiber bioreactors may offer greater process control. Controlling the environmental parameters within the bioreactors is one main technical challenges, however, controlling such parameters can mean that the phenotype of the cell-derived exosomes does not change in structure or functionality. When moving from static, planar cultures at the lab scale to a dynamic production-scale environment, the generation of shear stress from the impellers in the bioreactor and the cavitation of bubbles from oxygen sparging are still issues that may change the exosome product [204].

A bioreactor system should be designed to support adequate mass transfer in the cell culture. The reactor system can retain the exosome product within the culture condition medium to yield a more concentrated cultured medium, thereby reducing further downstream liquid handling requirements [206]. Owing to the poor production efficiency of exosomes in vitro, standard batch mode manufacturing can involve more expensive and complicated multilayer flask systems. Thus, it can take time to establish reproducible culture conditions and achieve robust production of exosomes with lot-consistent populations when scaling-up systems. Many basic environmental conditions and scale factors differentiate the culture media in small-scale flask- and impeller-based bioreactors. Culture progression and production efficiency-related concerns exist in relation to mass transfer differences, microcarrier binding, and hydrodynamic forces generated in sparging and agitation. Variations in these factors between bench and production scales can affect the culture characteristics in terms of the apoptosis potential, quality, and quantity of the exosome product [230]. The isolation methods used for exosomes in large-scale formats begin with tangential flow fractionation, which is frequently used as a first diafiltration and concentration step. Following TFF, the exosomal product is further purified by ultracentrifugation or other purification steps such as bead elute chromatography. Ultracentrifugation is difficult to scale up, while bead elute chromatography is relatively scalable [1].

The purification methods used in exosome production rely on the differences in size or density to purify specific cell-derived exosome populations or specific drug-loaded exosome populations.

Over the past few years, the development of exosomes has advanced towards clinical translation, while concerns related to isolation, storage, and GMP production remain challenging. During large-scale production, satisfactory yield of exosomes with good purity has been difficult to achieve, such that the optimal scaling-up conditions for therapeutically relevant exosomes need to be thoroughly evaluated.

## 10. Exosomes in Clinical Trials

Although exosome-based clinical trial studies are ongoing, the use of exosomes for drug delivery applications may be limited due to the possibility of loading exosomes with sufficient amounts of drugs, their potential to retain drugs under systemic circulation conditions, and their capability to deliver drugs to the target cells of interest in a functional state [152]. Several clinical trial studies have been carried out in patients, verifying the clinical benefits. Dendritic-cell- and mesenchymal-cell-derived exosomes are the two main categories of exosomes that have been applied in clinical trials.

Several phase 1 and one phase 2 clinical trial have been performed to evaluate the safety of exosome-based antitumor and antibacterial vaccines. In a phase 1 trial, dendritic cells were isolated from patients with advanced melanoma and then pulsed with tumor antigen. Exosomes carrying the tumor antigen were purified and injected into patients via intradermal and subcutaneous administration. After the administration, exosomes were found to be tolerated for up to 21 months with a mild inflammatory reaction at the site of injection. One out of 15 patients showed specific melanoma antigen T-cell response and a reduction in tumor size [231]. In another phase 1 trial, exosomes derived from autologous dendritic cells pulsed with melanoma antigen gene peptides were administered to patients with non-small cell lung cancer. Exosomes were well tolerated after administration weekly for up to 4 weeks and one-third of patients exhibited melanoma antigen gene-specific T-cell responses [232]. A phase 2 trial evaluated exosomes derived from interferon γ dendritic cells loaded with major histocompatibility complex I/II-restricted cancer antigens after induction of chemotherapy in patients with inoperable non-small cell lung cancer without tumor progression. The primary endpoint of the study was that at least 50% of patients after chemotherapy cessation should show progression-free survival at 4 months. One patient experienced a grade III hepatotoxicity and seven patients exhibited stabilization for more than 4 months out of 22 patients who received the therapy, suggesting that the exosomes that are derived from dendritic cells may be safe and may promote T and NK cell responses in patients [233,234].

Clinical trials have also been conducted on the use of exosomes as diagnostic and prognostic biomarkers for cancer. Clinical trials are being conducted on plant-derived exosomes to carry therapeutic agents in the treatment of colorectal cancer (CRC) (NCT01294072). In one such trial, curcumin was administered orally using exosomes to improve its pharmacological properties. The phase 1 clinical trial demonstrated the effects of curcumin-loaded exosomes on the immune modulation and cellular metabolism of normal and colon cancer subjects diagnosed with CRC.

A phase 2 clinical trial (NCT01854866) tested the effects of tumor cell-derived exosomes loaded with different chemotherapeutic agents in treating malignant ascites and pleural effusion. The subjects were treated with chemotherapeutic drugs such as hydroxyl camptothecin, cisplatin, doxorubicin, and methotrexate loaded into exosomes by adding them to the culture medium of the H22 and A2780 cancer cell lines [235]. In recent years, there has been an interest in using exosomes as potentially viable vaccines for immunotherapy. The exosomes derived from dendritic cells are enriched with the components, which function as antigen-presenting entities [236]. Exosome-based clinical trials have been conducted on patients with lung cancer and melanoma. These clinical studies employed exosomes derived from clinical-grade dendritic cells, with the method relying on combinations of ultrafiltration and ultracentrifugation to isolate exosomes from dendritic cell culture supernatant [237]. In phase 1 clinical studies, the injection of dendritic cells derived from exosomes in melanoma patients was shown to be safe, with some tumor regression and long-term stabilization. Further, increases in circulating natural killer cells and natural killer group 2 member D-dependent functions were observed in melanoma patients after injecting dendritic-cell-derived exosomes [231,238]. Similarly, in a phase 1 clinical trial, patients with stage III/IV lung cancer showed the tolerance of dendritic-cell-derived exosomes, with long-term stabilization being achieved in 4 out of 12 patients. [239]. The preclinical data have shown that mesenchymal-cell-derived exosomes are safe and scalable to large and clinically relevant doses. However, the clinical use of mesenchymal-cell-derived exosomes is limited due to the lack of optimal protocols for exosome production, isolation, and storage, meaning cell culture conditions, administration schedules, and reliable characterization methods must be established to evaluate the efficacy of exosome therapy [240].

Nassar et al. evaluated the effects of mesenchymal-cell-derived allogeneic cord tissue exosomes on B-cell mass in patients with type 1 diabetes mellitus (NCT02138331). The preclinical trial prior to this clinical trial showed that mesenchymal-cell-derived exosomes prevented the onset of the disease in a mouse model of type 1 diabetes and effectively suppressed autoimmunity. The results indicated that exosome therapy suppresses the development of T helper cells (Th1 and Th2), restoring the balance between immunological responses of Th1 and Th2 [241,242]. The study results of this clinical trial have not yet been published. A phase 2/3 clinical trial was conducted using cord-tissue-derived MSC exosomes to improve the renal functions in patients with chronic kidney disease. The primary endpoint of the study was safety, while the secondary endpoint was improvements in the glomerular filtration rate or reductions in plasma creatinine levels. The exosome therapy resulted in an improved glomerular filtration rate and a decrease in the creatinine level [240,243,244]. The details of the clinical trials related to exosome-based drug delivery are presented in Table 6.

**Table 6:** List of clinical trials for exosome-based therapeutics.

Study Title	NCT Number	Conditions	Phase	Outcome Measures	Source of Exosomes
Allogenic mesenchymal stem cell derived exosome in patients with acute ischemic stroke.	NCT03384433	Cerebrovascular disorders	Phase 1/2	Incidence of treatment-emergent adverse events (deteriorating stroke, stroke recurrences, brain edema, seizures, hemorrhagic transformation). The degree of disability in stroke patients.	Mesenchymal stem cell
Evaluation of adipose derived stem cells exosomes in treatment of periodontitis.	NCT04270006	Periodontitis	Early phase 1	Changes in gingival inflammation, bone levels, probing depth.	Adipose-derived stem cells
Study investigating the ability of plant exosomes to deliver curcumin to normal and colon cancer tissue.	NCT01294072	Colon cancer	Phase 1	The concentrations of curcumin in normal and cancerous tissue, safety and tolerability of curcumin, and immune system response to curcumin.	Plants (fruit)
Effect of plasma derived exosomes on cutaneous wound healing.	NCT02565264	Ulcers	Early phase 1	Ulcer size, the pain of cutaneous wounds.	Plasma
Trial of a vaccination with tumor antigen-loaded dendritic cell-derived exosomes.	NCT01159288	Non-small cell lung cancer	Phase 2	Progression-free survival.	Dendritic cells
Edible plant exosome ability to prevent oral mucositis associated with chemoradiation treatment of head and neck cancer.	NCT01668849	Head and neck cancer and oral mucositis	Phase 1	Pain caused by oral mucositis, levels of immune biomarkers in blood and mucosal tissue.	Plants (grape)
Effect of microvesicles and exosomes therapy on β-cell mass in type I diabetes mellitus.	NCT02138331	Diabetes mellitus type 1	Phase 2 and phase 3	Total daily insulin dose, pancreatic β-cell mass, and hemoglobin A1c.	Mesenchymal stem cells
Exosomes and Immunotherapy in Non-Hodgkin B-cell lymphomas.	NCT03985696	Lymphoma, B-cell, aggressive non-Hodgkin	Not applicable	Quantification of CD20 and PDL-1 in exosomes purified from cell cultures of diffuse large B-cell lymphoma (DLBCL) human cell lines and evaluation of whether peripheral exosomes can be used as novel diagnostic biomarkers in DLBCL.	Tumor B cells
Exosomes in treating participants with metastatic pancreas cancer with KrasG12D mutation.	NCT03608631	Metastatic pancreatic adenocarcinoma Pancreatic ductal adenocarcinoma Stage IV pancreatic cancer AJCC v8	Phase 1	Overall survival, progression-free survival, minimal residual disease rate in high-risk patients, and maximum tolerated dose determined by dose-limiting toxicity.	Mesenchymal stromal cells
A safety study of IV stem cell-derived extracellular Vesicles (UNEX-42) in preterm neonates at high risk for BPD.	NCT03857841	Bronchopulmonary dysplasia (BPD)	Phase 1	Safety and tolerability, incidence, and severity of BPD at 36 weeks postmenstrual age and incidence of death at 36 weeks postmenstrual age.	Bone marrow mesenchymal stem cells

## 11. Regulatory Challenges

Native exosomes contain a huge number of proteins and nucleic acid components, which may have intrinsic therapeutic effects, making the exosomes themselves biological products. Although the use of exosomes has been proven clinically, no exosomal products have been approved by the regulatory agencies [230]. Exosome therapy is considered a subset of cell therapy. Regulations similar to those used for biologics products will be required to ensure that the manufacturing process for exosomes complies with GMP requirements, while the components used in manufacturing should be qualified to assure the biological activity of exosomes. Furthermore, the characterization of the cell sources used to isolate the exosomes is critical and donor eligibility criteria must comply with the requirements of the regulatory framework. To ensure the safety of exosome-based therapeutics, issues related to cell bank qualification and product quality need to be addressed. In cell bank testing, the characterization of essential steps is needed in order to obtain a uniform product with lot-to-lot consistency and to demonstrate that the cell lines that are used to isolate the exosomes should be free from endogenous viruses and other adventitious agents. The presence of contaminants, including host cell DNA and extraneous soluble proteins, may result in increases in the risk of immunogenic responses and adverse side effects, compromising product quality [245]. Therefore, a complete understanding of the characteristics and molecular composition of each exosome type, their impurity profiles, and their harvest sources is needed, while the manufacturing and isolation of exosome-containing products should follow a set of applicable quality and regulatory considerations, as with many other biological products [230]. Although no regulatory agencies or national compendia have established guidance or standards, the USFDA recently approved an investigational new drug to begin clinical trials for exosomes isolated from bone-marrow-derived mesenchymal stem cells to treat severe dermatological conditions in burn patients [246]. Despite the biological and regulatory complexity of exosome-based therapeutics, it is necessary to develop scalable and reproducible purification protocols using a robust risk-based approach to produce exosomes of desired quality. The regulatory agencies, including the EMA and USFDA, consider human exosome-based therapeutics as biologic products. Exosome therapeutics manipulated pre- or postisolation, including through expansion of parent cells or genetic manipulation, are classified as advanced therapy medicinal products (ATMPs) [247]. Compared to mesenchymal stem cell (MSC) therapies, exosome-based therapeutics have the potential to overcome safety concerns related to MSC proliferation while maintaining similar therapeutic effects, which could be due to the lower risk associated with exosomes than cell-based therapeutic approaches. However, the limited number of clinical trials on exosome-based therapeutics depict the problems associated with understanding the molecular functions generated by exosomes in target cells [248]. There are still many hurdles to overcoming the complex nature of exosome-based therapeutics, including determining the route of administration, frequency of treatments, optimal dose, and time window for exosome delivery to accomplish maximum efficacy without any adverse effects. Safety standards relevant to the cell source or conditioned medium are one important parameter for the characterization of exosome-based products in both non-clinical and clinical applications [249].

## 12. Future Prospects and Concluding Remarks

The exploitation of exosome vesicles as drug delivery systems is highly dependent on the cell source and type. Because of the ability of exosomes to transfer therapeutics to recipient cells via an endogenous uptake mechanism, the exosome vesicle is a promising candidate for drug delivery. Nevertheless, current obstacles related to scalable exosome isolation methods and efficient drug loading approaches and guidelines for appropriate storage must be overcome before exosomes can be used at the production scale in clinical trials. The long-term storage stability of exosomes is one important issue that needs to be further investigated. Studies have shown that drying techniques such as lyophilization using trehalose could prevent exosomal damage by preserving the exosomes’ endogenous content (protein and RNA). Another important challenge in exosome drug delivery is to ensure that exosome-based therapeutics meet the requirements of the regulatory bodies to ensure clinical approval. Compared to other types of nanomedicines, the regulatory considerations for exosome-based therapeutics have not been tackled thoroughly to date. Although research and clinical studies related to the use of exosome-based therapeutics for drug delivery are still in their infancy, the on-demand methods used for advanced understanding and systemic characterization of exosomes will address the challenges and clinical transition issues of exosome-based therapeutics.

## Data Availability

The study did not report any data.
